# The Gene Expression Program for the Formation of Wing Cuticle in Drosophila

**DOI:** 10.1371/journal.pgen.1006100

**Published:** 2016-05-27

**Authors:** Lukasz F. Sobala, Paul N. Adler

**Affiliations:** Biology Department and Cell Biology Department, University of Virginia, Charlottesville, Virginia, United States of America; The University of North Carolina at Chapel Hill, UNITED STATES

## Abstract

The cuticular exoskeleton of insects and other arthropods is a remarkably versatile material with a complex multilayer structure. We made use of the ability to isolate cuticle synthesizing cells in relatively pure form by dissecting pupal wings and we used RNAseq to identify genes expressed during the formation of the adult wing cuticle. We observed dramatic changes in gene expression during cuticle deposition, and combined with transmission electron microscopy, we were able to identify candidate genes for the deposition of the different cuticular layers. Among genes of interest that dramatically change their expression during the cuticle deposition program are ones that encode cuticle proteins, ZP domain proteins, cuticle modifying proteins and transcription factors, as well as genes of unknown function. A striking finding is that mutations in a number of genes that are expressed almost exclusively during the deposition of the envelope (the thin outermost layer that is deposited first) result in gross defects in the procuticle (the thick chitinous layer that is deposited last). An attractive hypothesis to explain this is that the deposition of the different cuticle layers is not independent with the envelope instructing the formation of later layers. Alternatively, some of the genes expressed during the deposition of the envelope could form a platform that is essential for the deposition of all cuticle layers.

## Author Summary

Insects and other arthropods are an extremely successful group of animals. A unique and key feature of their lifestyle is their chitin containing cuticular exoskeleton, a complex layered material, which remains rather poorly understood for so prominent of a biological material. We have characterized the gene expression pattern of wing epithelial cells over the period of cuticle formation and also carried out transmission electron microscopy, which allows us to identify genes that likely play a role in the formation of different cuticle layers. Functional studies suggest that the deposition of the earliest layer influences the deposition of the later ones.

## Introduction

The cuticular exoskeleton of insects provides multiple functions for the animal. It provides shape, protects the animal from the environment, limits water loss and provides the skeletal elements needed for locomotion. In different tissues of an individual animal the cuticle will have very different physical properties. For example, in one region the cuticle can be stiff and hard while in another region soft and elastic. The Young's modulus of different cuticles varies over about 8 orders of magnitude–a greater range than any other biological material [1]. The structure of the cuticle is as varied as its function and physical properties and is composed of multiple layers [1–3]. Cuticle is secreted in a sequential fashion by epidermal cells with the most external layer being secreted first. The number of layers and their thickness varies from one cuticle to another and insect cuticle has served as inspiration for a variety of human engineered material [4–6].

Insect cuticle contains proteins, chitin, lipids and water all of which are thought to be functionally important [1]. Some cuticle proteins have been identified by analyzing extracted soluble proteins from mature cuticle or cuticle in the process of synthesis [7–9]. Others have been identified by the analysis of RNA in cuticle synthesizing cells, by comparing the sequence of known cuticle proteins to genome sequences [10–13] or by serendipity [14]. The number of cuticle proteins encoded by the Drosophila genome is estimated to be around 150 and in some insect genomes the estimate is more than 200 [15]. A number of other proteins have been identified as being essential for the synthesis of normal cuticle such as chitin synthase [16, 17], chitinases [18, 19], Knk [20, 21] and ZP domain proteins [18, 22–24]. It is unclear if any of these ends up being part of the cuticle proper, although that has been suggested for ZP domain proteins [22].

The analysis of cuticle composition is complicated by the cross linking that is part of cuticle maturation [25–27], the insolubility of some components and because other tissues and cell types become attached to the cuticle (e.g. muscles) as it is being synthesized by epidermal cells. The wing provides potential advantages as pupal wings can be dissected in a pure form without attached muscle or other tissues. We have taken advantage of this and characterized the pupal wing transcriptome of Drosophila by RNAseq [28, 29]. We also examined pupal wings by transmission electron microscopy (TEM) over the period covered by the RNAseq experiments allowing us to correlate the dramatic changes in gene expression with the morphological process of cuticle deposition. The expression of many genes was almost completely restricted to a single time point making these genes candidates for taking part in the deposition of specific cuticle layers.

Mutations in a number of genes expressed almost exclusively in 42 hr pupal wings are known to result in dramatic wing phenotypes (e.g. *miniature* (*m*), *dusky* (*dy*) [22] and *dusky-like* (*dyl*) [18]). This time point is in the middle of the period when the thin outermost envelope layer is deposited. It is known that wing procuticle, which is synthesized much later, is grossly abnormal in a *m dy* double mutant [22] and we here found that to also be the case for *dyl*. We also identified wing and cuticle phenotypes of additional genes primarily expressed at 42 hr and for two of these transmission electron microscopy established that there is a procuticle phenotype. To explain the extent of the mutant phenotypes seen with a loss or decrease in the function/expression of “42 hr genes” we suggest either that the successively laid down layers are not independent and that earlier layers instruct the deposition of later ones or that some of the “42 hr genes” form a platform or complex that mediates the deposition of all of the cuticle layers.

## Results

### The time course of wing development and cuticle deposition

**Fig 1:**
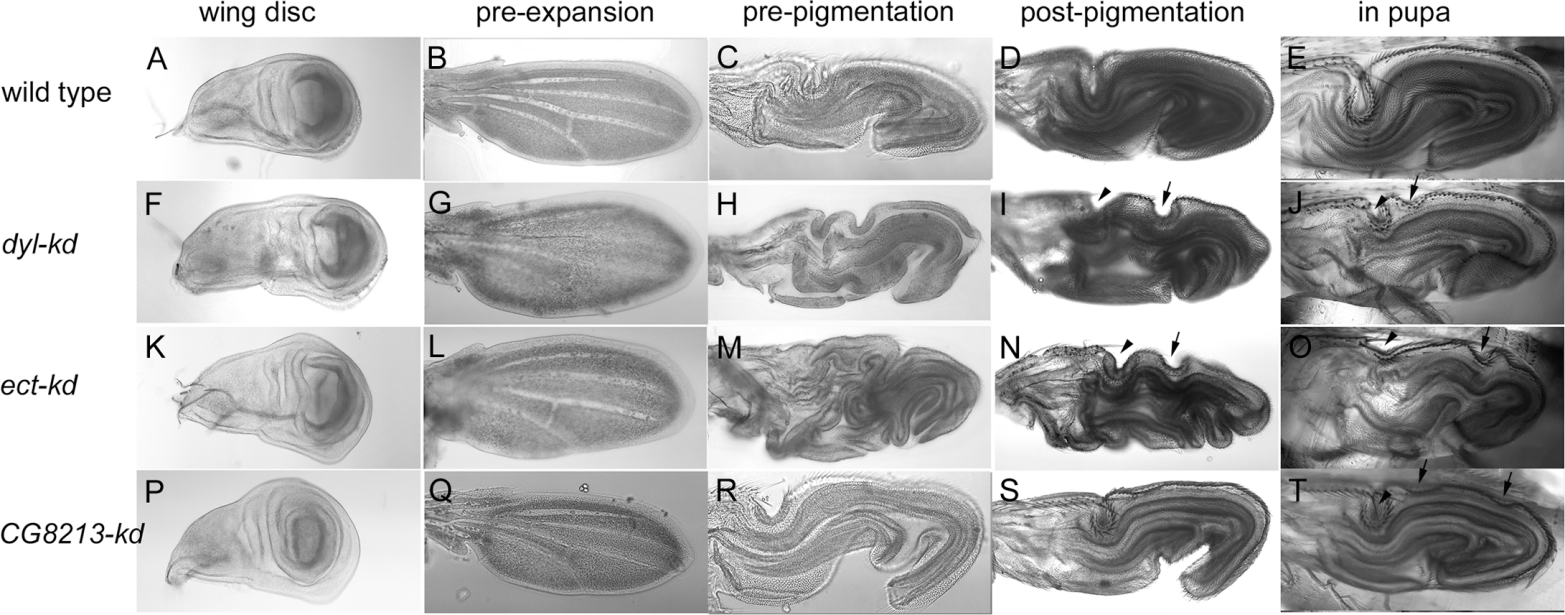
Changes in pupal wing morphology over time in wild type and mutants. Shown are wing discs and pupal wings from wild type (Ore-R (A-E), *dyl*-kd (F-J), *ect*-kd (K-O) and *CG8213*-kd (P-T) animals. The wing discs were dissected from wandering third instar larvae and during this period the disc prepares for evagination. This leads to some discs being a bit more 3 dimensional (this can be seen in panel F). The folding pattern seen in post-flattening/expansion wild type pupal wing discs is highly reproducible. The mutants are more variable from animal to animal. This was particularly true for *ect*, where males showed a stronger phenotype than females. The final column shows pigmented pupal wings imaged through the pupal case and establishes that the differences between wild type and mutants are not due to dissection. The arrowheads point to shallower than normal proximal anterior folds and the arrows to ectopic folds seen in mutants. Note that during the process of dissection there is often some relaxation in the folding pattern of younger (pre-pigmentation) pupal wings.

The Drosophila wing tissue undergoes a number of dramatic morphogenetic transitions during development [18, 30]. The wing and the dorsal thorax are derived from the wing disc (Fig 1A) that proliferates throughout most of the larval period. The wing disc undergoes evagination after the start of pupariation producing a pupal wing that is a small version of the adult wing (Fig 1B). All cell division in the wing ceases around 24 hr after white prepupae (awp). The terminal differentiation of the Drosophila wing begins around 32 hr awp when the morphogenesis of the wing hairs (trichomes) starts [31]. The start of cuticle deposition is seen by the middle of wing hair outgrowth (~36 hr awp)[31–33]. At this stage diffuse material and small patches of envelope are seen by transmission electron microscopy (TEM). Around 40 hrs awp hair outgrowth is largely complete. Around 40–42 hr awp the wing cells begin to flatten and increase their apical surface area (Fig 1C and S1A Fig)(18) a process we refer as flattening/expansion. Since the wing is enclosed within a pupal cuticle sac it is forced to deform and bend back on itself (Fig 1B–1E, S1A–S1E Fig)(18). In previous experiments we first observed chitin in hairs around 42 hr awp and later in wing blade cuticle [18, 34]. Pigmentation of the wing was first obvious around 80 hr awp (Fig 1D and 1E, S1C and S1D Fig) and eclosion of the adult was around 96 hr awp. After eclosion the wing unfolds and extends to take on the shape of the adult wing (S1E Fig).

**Fig 2:**
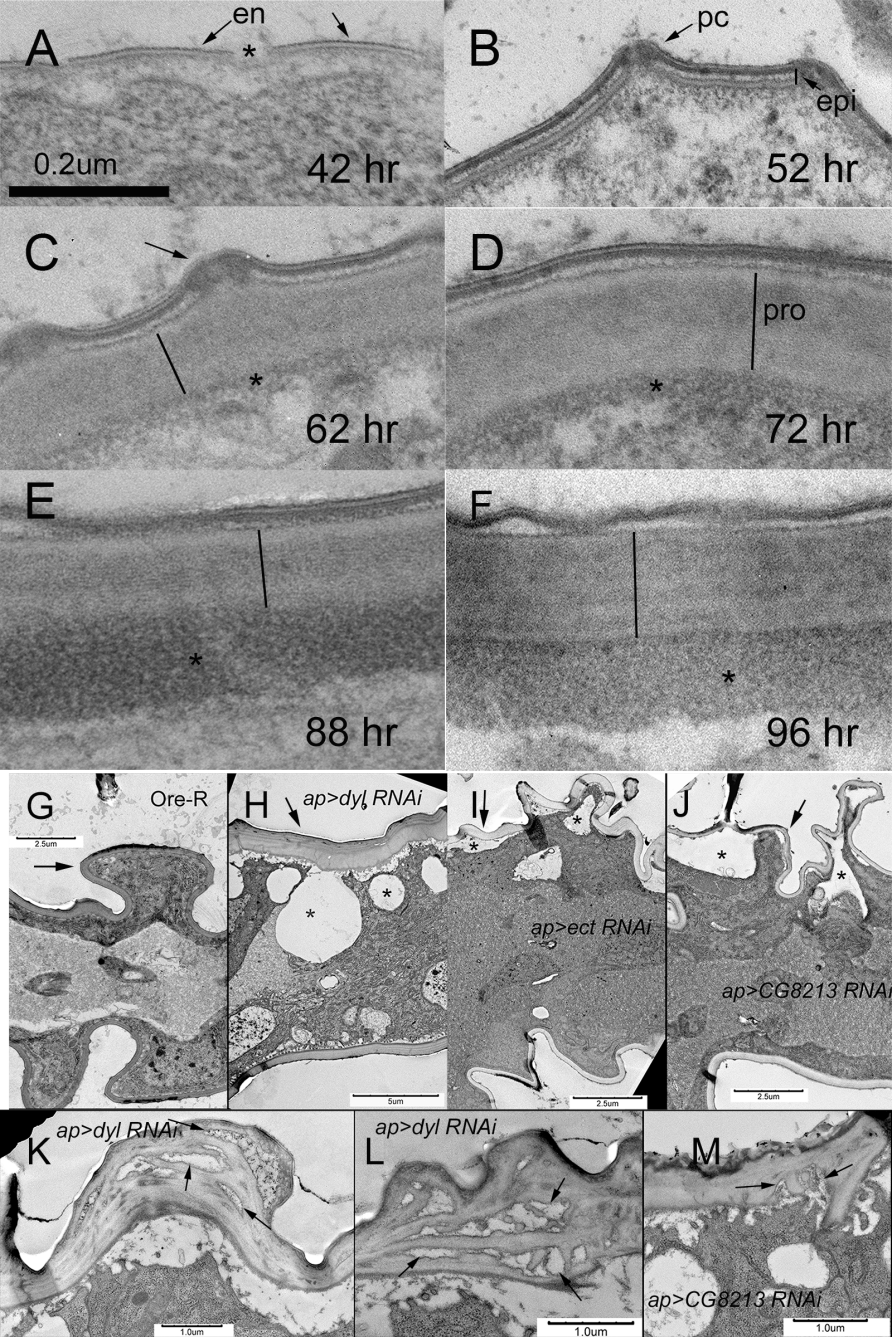
Transmission electron micrographs of wing cuticle deposition. Panels A-F are all shown at the same magnification. The time is for pupae collected as white prepupae and aged at 25°C. Panel A. In 42 hr awp wings the deposition of the envelope (en) is visible (arrows). Gaps remain where the envelope does not cover the surface (asterisk). B. In 52 hr awp wings the epicuticle (epi) is now visible (line) and pore canals are prominent (arrows—pc). C. In 62 hr wings procuticle is now obvious (line). Pore canals remain prominent (arrow) and at the base of the procuticle a dark granular layer is first seen (asterisk). This may be the adhesion layer. D. In 72 hr wings the procuticle (pro) is prominent (line) and the dark granular layer is more obvious (asterisk). Panel E. In the 88 hr wing the procuticle (line) and dark granular layer (asterisk) are both prominent. Panel F. In the 96 hr wing the procuticle (line) and dark granular layer (asterisk) are both prominent. Panels G-J are relatively low magnification electron micrographs of 76–80 hr pupal wings where both the dorsal and ventral cuticle can be seen. In all of these images the putative dorsal surface is marked by an arrow. G. Ore-R. H. *ap>dyl RNAi*. I. *ap>ect RNAi*. J. *ap>CG8213 RNAi*. Note in all three of these images there are gaps between the apical surface of the dorsal wing cells and the cuticle (asterisks) and the dorsal and ventral cuticle is of different thickness in H-J compared to G. In panel G the arrow also points to a hair pedestal. Panels K and L show regions of highly abnormal cuticle in *ap>dyl RNAi* wings. The arrows point to gaps/holes in the procuticle. M shows a region of an *ap>CG8213 RNAi* wing with abnormal procuticle (arrows).

Early evidence for the beginning of cuticle deposition can be seen in 35–38 hr pupal wings. In these wings we observed disorganized relatively electron dense material close to the apical cell membrane (S2 Fig, arrowheads). Over the wing blade region of cells it was rare to see evidence of the trilaminar envelope. It is possible the amorphous material is a precursor to the envelope. Cuticle deposition appeared somewhat more advanced over the hairs, as we often saw patches of envelope there (S2 Fig, arrows). In wings 42 hr awp we observed the trilaminar envelope over much of the epidermal cells (Fig 2A, arrows) and hairs but it was not continuous and gaps were present (Fig 2A, asterisks). Similar observations were made on 45 and 48 hr awp pupal wings although the gaps appeared less frequent. In 52 hr awp pupal wings the envelope was continuous and a layered epicuticle was seen (Fig 2B). Pore canals were observed in the 52 hr wings (Fig 2B, arrows) and we often observed what appeared to be material being secreted from these pores. In both early stages and later ones we often observed protrusions of the cytoplasm in close juxtaposition to the cuticle (S2A Fig and S3A and S3B Fig, arrows) as has been seen previously (e.g. [22, 35]). These are thought to represent sites of cuticle deposition. Although they appear as rectangles in these sections they are likely rows of elevated cytoplasm equivalent to the undulae described for the deposition of the first instar larval cuticle [35]. The procuticle layer appeared relatively thick by 62 hrs with additional thickening over time (Fig 2C–2F), although differences in the angle of sectioning can be misleading as to thickness. At later stages the layered nature of the envelope and epicuticle was less obvious and subtle layering in the procuticle was more obvious (e.g. 96 hr awp) (Fig 2F). We refer to this cuticle maturation and it implies that some late gene expression modifies the structure of the cuticle layers deposited earlier. Filaments were seen in the procuticle and these are likely chitin. There was also a thin basal electron dense layer in 62 hr wings (Fig 2C, asterisks) that appears similar to the adhesion layer seen during the synthesis of the first instar larval cuticle [17, 36] and perhaps what was called Schmidt’s layer in older studies on cuticle [37, 38]. This putative adhesion layer thickened and became more electron dense over time and was very prominent in 88 and 96 hr wings (Fig 2E and 2F, asterisks). This corresponds to the principal period of wing pigmentation and while there is relatively little pigmentation in the Drosophila wing blade compared to hairs it is possible that the material observed in 88 and 96 hr wings is pigmented procuticle. Thus, by morphology we can distinguish periods for envelope deposition (36–48 hr awp), epicuticle deposition (52 hr awp), procuticle thickening (62–88 hr awp), and the period of cuticle maturation and thickening of the adhesion layer/pigmentation of basal procuticle (88–96 hr awp).

Due to possible differences in the angle of orientation we did not feel it justified to compare the thickness of cuticle from different sections or time points. In relatively low magnification TEM images (3–5,000X) we could often observe both dorsal and ventral cuticle and cell layers (Fig 2G). The two cuticle layers appeared to be relatively similar in thickness. However, when we measured the thickness of the layers (see Methods) we observed that one layer was reproducibly thicker (ave ratio = 1.2 (sd = .11)) (S1 Table). We suspect the dorsal surface is the thicker one as dorsal hairs are larger than ventral ones.

As noted above hair cuticle deposition appeared slightly advanced compared to wing blade cuticle. By 52 hrs awp the developing hairs were found on pedestals (S3H Fig, arrow and had taken on their fluted shape (S3D–S3H Fig). The pedestals are bulges of cytoplasm covered by cuticle that does not differ dramatically from that of the remaining wing blade cuticle (40). One difference is the cuticle in the center of the pedestal often appears darker by TEM. This could be due to pigment as is seen in the hairs. The size of the hairs in the micrographs was dependent on where along the apical basal axis of the hair the section was cut. During the remainder of pupal development the internal morphology of the hair changed dramatically with both electron dense and lucent regions seen (S3D–S3F Fig). At later stages (e.g. 88 hr, 96 hr) it was primarily electron dense, perhaps due to pigmentation of the hair (S3 Fig). One difference between the formation of hair and wing blade cuticle is that distinct undulae were infrequently associated with hair cuticle (see also [33]). Overall our TEM observations are consistent with those by others (e.g. [33, 39, 40]) although a complete TEM time series of wing cuticle deposition has not to our knowledge previously been presented.

### Gene expression during cuticle deposition

**Fig 3:**
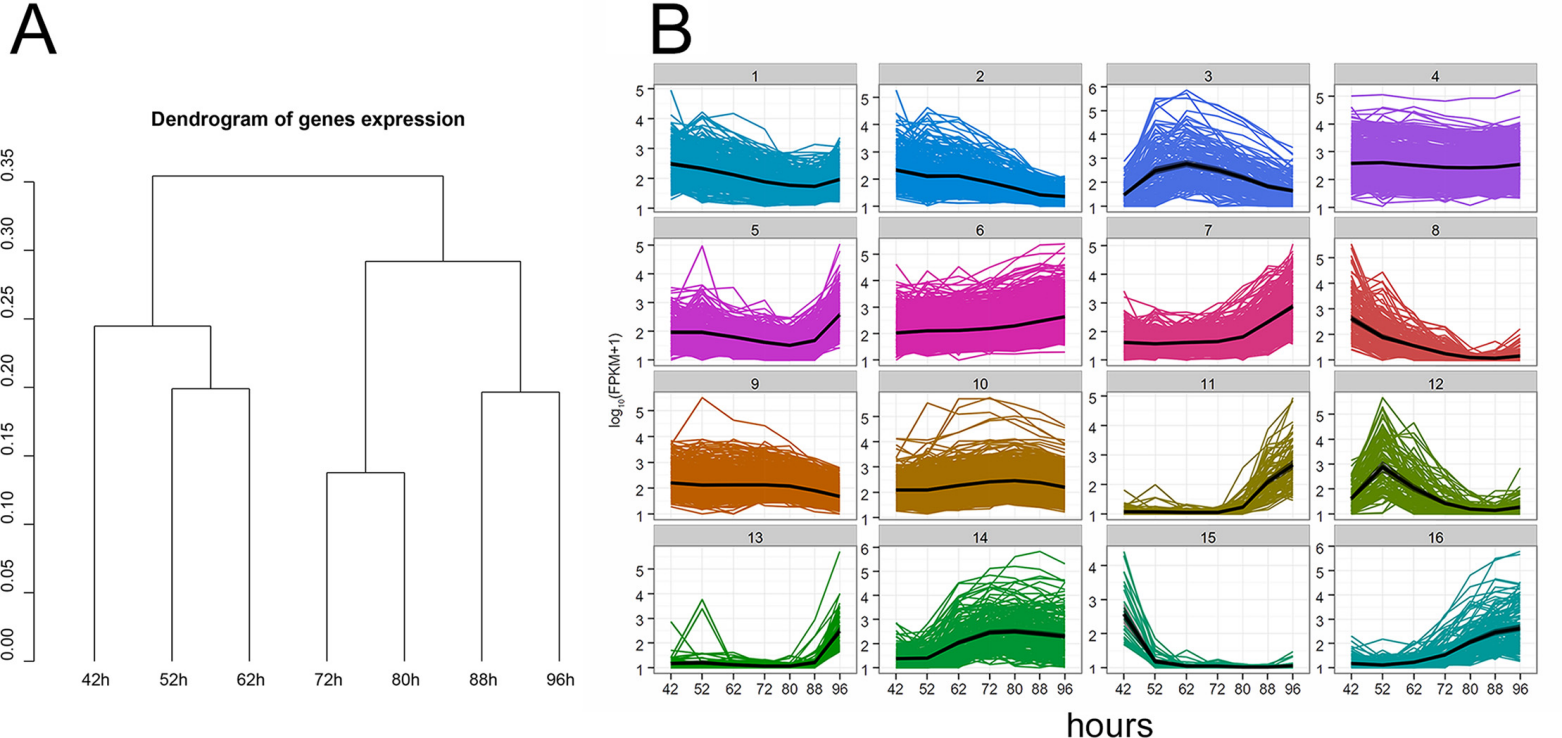
Gene expression clusters during the deposition of wing cuticle. A. The dendrogram shows the relationships between the gene expression patterns at different time points. The scale refers directly to the mutual Jensen-Shannon distance between branches. B. The gene expression patterns for all of the 16 clusters. The numbers on the Y axis show the log_10_(FPKM+1) values and the X axis shows the time. The graphs show the expression patterns for the members of each of the 16 clusters. Clustering methods are described in more detail in S1 File (Supplementary Methods). Colored lines represent the pattern for each gene and the black line represents the medoid.

We characterized the transcriptome of pupal wing cells at 42, 52, 62, 72, 80, 88 and 96 hrs awp by RNAseq (S2 Table). The last time point was just prior to eclosion. 9,088 out of 17,243 genes were expressed in at least one of the time points using the criteria that anything with an FPKM (Fragments Per Kilobase of exon per Million reads) greater than 1 was expressed (S2 Table). Clustering of the gene expression data based on mutual Jensen-Shannon divergence (Fig 3A) indicated that the 3 early time points clustered together as did the 4 later time points. The clustering of the 52 and 62 hr samples was surprising as morphologically the 62 hr wings appeared more similar to the 72 hr wings. There was a trend where the number of differentially expressed genes increased when we compared more distantly separated time points (S3 Table). In many cases the changes in gene expression were quite large (e.g. there were more than 100 cases where a 100 fold or larger difference was seen between neighboring time points—Table 1). 5097 individual genes showed a statistically significant (q<0.05) difference in expression between at least one pair of time points (S4 and S5 Tables). The largest number of differences were seen between the 42 and 52 hr wings and the 88 and 96 hr wings (Table 1) while the smallest number were seen between the 72 and 80 hr wings.

**Table 1:** Number of genes whose expression level changed between time points.

Time1	Time 2	# differences	>10 fold	>100 fold
42 hr	52 hr	1624	334	70
52 hr	62 hr	1266	211	16
62 hr	72 hr	999	99	19
72hr	80 hr	722	65	1
80 hr	88 hr	1013	125	3
88 hr	96 hr	1639	270	31

**Fig 4:**
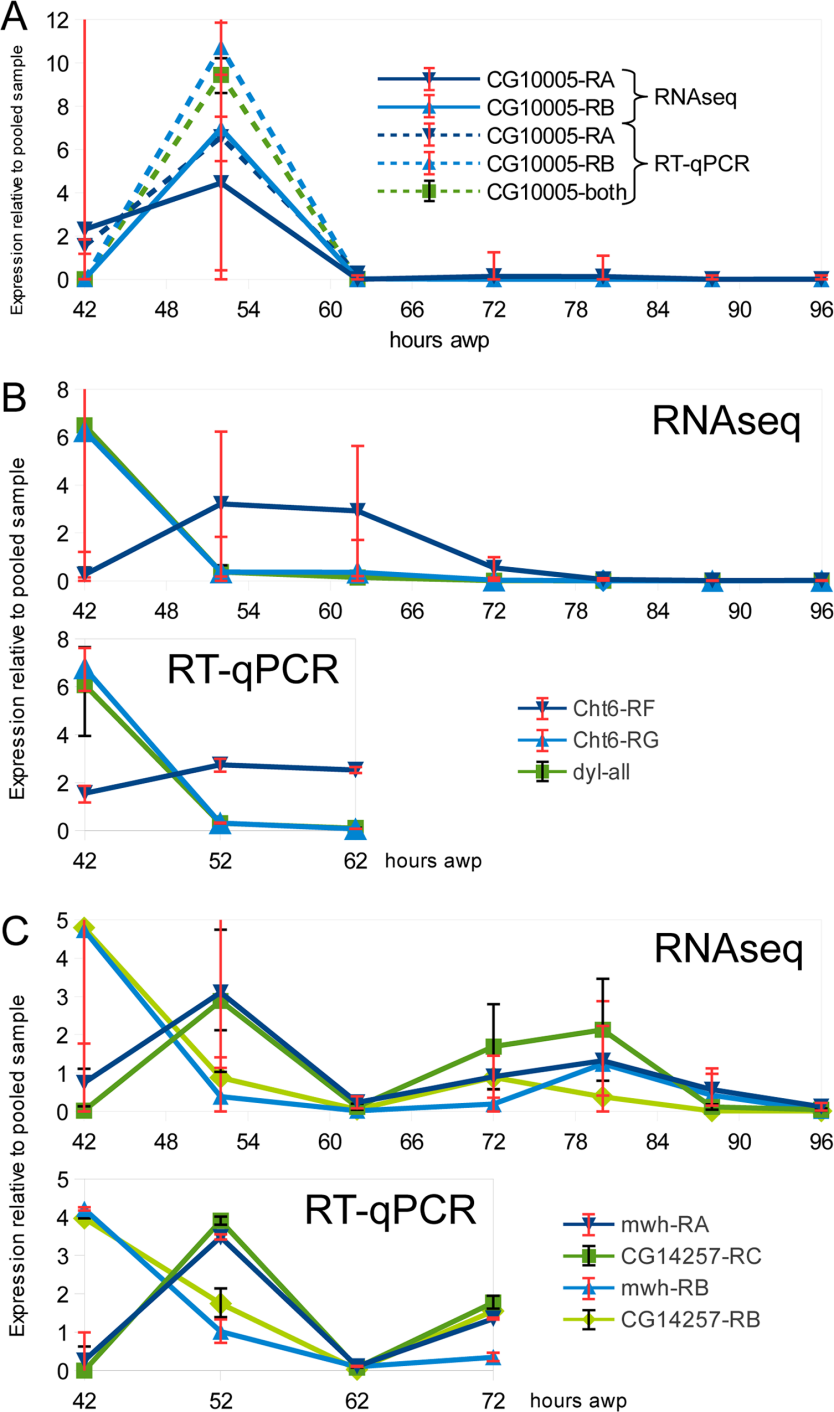
Similar results are obtained by both RNAseq and RT-qPCR. A. The relative gene expression patterns for the two mRNAs encoded by the CG1005 gene. Note the similar pattern seen with both RNAseq and RT-qPCR. B. The expression pattern for *dyl* and two isoforms of *Cht6*. Note that Cht6-RG expression follows a similar pattern to *dyl*. C. The expression patterns for two isoforms of *mwh* and CG14257 show qualitatively similar changes when assayed by both RNAseq and RT-qPCR. Note that relative expression values as a function of time and not absolute expression values are shown in this figure.

As a control that the expression differences were not dependent on the approach we carried out RT-qPCR Relative Quantification (RQ) for a pair of ZP domain genes whose expression was highly stage specific (*dyl* and *CG10005*) Our endogenous control was *Xbp1*, a gene that was rather evenly expressed in our RNAseq data (FPKM around 100). A similar temporal pattern of expression was detected in both the RNAseq and RT-qPCR experiments (Fig 4A). As is discussed later we also examined the data for changes in specific isoform usage.

We took an alternative approach to identify genes expressed in a highly stage specific manner. For this we added up the FPKM values for each gene over the 7 time points and identified genes where expression was primarily seen at one time point (S6 Table). The 42 and 96 hr samples stood out by having many genes (67 and 87 respectively) where >90% of their total FPKM was found in that sample while there were no such genes in the 80 and 88 hr samples and only 1 in the 72 hr sample.

### Gene expression clusters

The 5097 genes that showed expression differences were placed into 16 clusters (Fig 3B and S5 Table). The clusters contained from 31–860 genes and each contained genes with a wide variety of absolute expression values. Ten of the clusters (3, 5, 7, 8, 11, 12, 13, 14, 15 and 16) displayed dramatic stage specific changes in expression patterns. These clusters were greatly enriched for genes where homologs are only found in arthropods (average: 53%, range: 28–94%) compared to the genes present in the other clusters (average: 19%, range: 15–27%) (S7 Table). This difference was significant (t-test, p = 1.25X10^-4^). This suggests these clusters are enriched in genes involved in cuticle deposition. Among the clusters showing the most dramatic changes in gene expression were cluster 15 (highly expressed only at 42 hrs) and cluster 13 (highly expressed only at 96 hr).

We expected this set of 5097 genes contained many required for normal wing development. When we queried FlyBase for genes associated with wing phenotypes we found 2611 such genes. A majority of these (1469 (56%) were present in the differentially expressed gene set (S7 Table). The frequency of annotated wing phenotypes was not similar in all of the clusters (S7 Table). Similarly, the frequency of genes identified in a genome wide RNAi screen [41] was not similar in all clusters S6 Table).

We observed both under and overrepresentation of Gene Ontology associations in the clusters (S8 Table). For example, clusters 4, 5, 6, 9 and 10 contained a high proportion “housekeeping” genes taking part in cellular respiration, replication, translation and protein turnover. Cluster 4 was the only one enriched in “metabolic” genes and the only one in which “structural components of chitin-based cuticle” were underrepresented. The best structure was found in clusters 15, 4 and 11 (cluster silhouette >0.2). In clusters 3, 8 and 11–16 genes connected with chitin-based cuticle were overrepresented and there was a high proportion of arthropod-specific genes.

### Expression patterns of cuticle protein encoding genes and cuticle related genes

**Fig 5:**
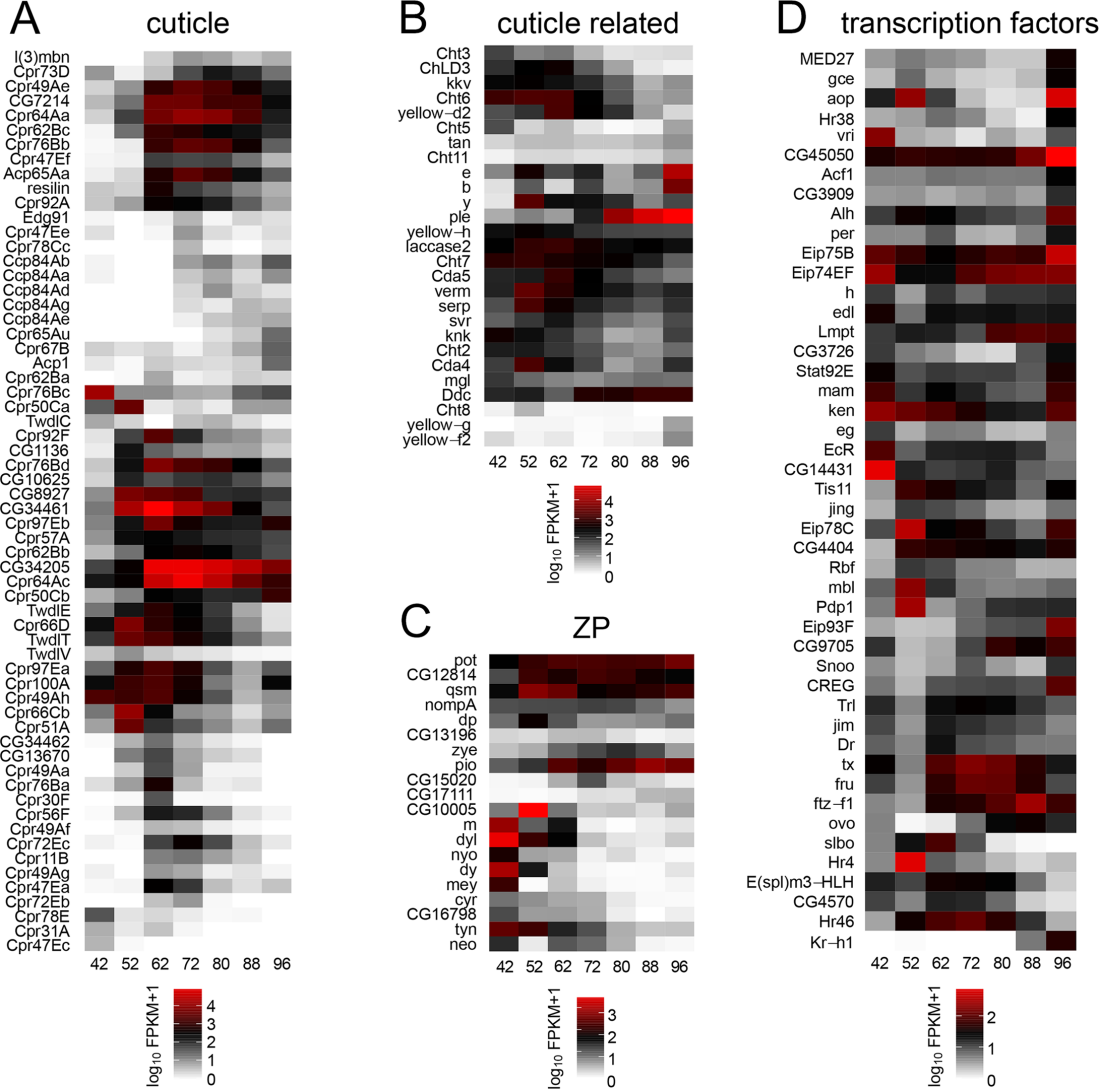
Heat maps for 4 groups of related genes. A. Annotated cuticle proteins. B. Genes with a known function in cuticle deposition or maturation. C. ZP domain protein encoding genes. D. Genes that regulate transcription.

We found 146 genes in fly genome annotated as encoding cuticle protein genes. Eighty three of these were expressed in the pupal wing using the criteria that there was at least one time point with an FPKM >1 (S9 Table). Some of these genes were expressed at a very high level. For example, 25 had a total FPKM of greater than 1000. There were a variety of expression patterns for these genes (Fig 5A) and some were expressed primarily at a single time point and others were expressed at a high level in all of the time points. For example, Cpr76Bc was expressed at a very high rate in 42 hr pupal wings but not at other stages, suggesting it might function in envelope morphogenesis. A recent RT-PCR analysis of selected cuticle proteins in Bombyx mori during pupal cuticle formation also found examples of very sharp expression peaks [42]. Over 97% of the total FPKM for annotated cuticle proteins was contributed by just 22 genes and more than 70% was contributed by just 3 (CG34205, Cpr64Ac and CG34461). Thus, if cuticle protein content was reflected in mRNA content and the annotated cuticle proteins represented the true population of cuticle proteins, the protein content of wing cuticle on a mass basis would not be as complex as the number of cuticle protein genes suggests it might be.

In addition to cuticle protein genes a number of other genes expressed at relatively high and varying levels in pupal wings are known to be important for normal cuticle deposition (Fig 5B). These include genes such as *krotzkopf verkehrt* (*kkv*) ([16, 43](cluster 2), which encodes chitin synthase and *knickkopf* (*knk*) [20, 21, 43](cluster 1), which protects chitin from degradation. We also found 5 chitinases and a number of genes involved in pigmentation and sclerotization expressed. Among the most notable were *pale* (*ple*) [44] (cluster 16)), *ebony* (*e*) [45] (cluster 5), *Dopa decarboxylase* (*Ddc*) [46, 47](cluster 6), and *yellow* (*y*) [48, 49](cluster 3).

### Expression patterns of ZP domain protein encoding genes

A number of ZP domain proteins are essential for cuticle formation in both the embryo and pupae [18, 22–24]. Eighteen of the 20 fly ZP domain protein genes were expressed during wing cuticle deposition. The pattern of expression was dynamic and differed greatly between ZP domain genes. Several genes: *dusky-like* (*dyl*), *dusky* (*dy*), *miniature* (*m*), *trynity* (*tyn*), *morpheyus* (*mey*), *nyobe* (*nyo*) were expressed at a much higher level at 42 hr than at any of the other time points (Fig 5C). All of these genes function in the formation of denticles in the embryo [23] and *dyl*, *dy* and *m* mutants have dramatic wing phenotypes [18, 22]. These data support the idea that these proteins function in the deposition of the cuticle envelope, as was suggested some years ago by Roch et. al. for *m* and *dy* [22]. Other ZP domain protein encoding genes are primarily expressed at other stages. *CG10005* (cluster 12) stood out because it was expressed at a much higher level in 52 hr wings than at any other stage, suggesting it functions in epicuticle deposition. Only the shorter transcript of this gene was expressed in pupal wings. Its associated polypeptide (182 aa) consists almost entirely of the ZP-N, which is evolutionarily more conserved than ZP-C. Most ZP domain proteins contain both sub-domains. In experiments described previously we found that knocking down *Cht6* expression produced a weaker version of the *dyl* mutant wing hair phenotype and these two genes showed a positive genetic interaction [18]. The expression pattern of *Cht6* did not closely resemble that of *dyl*, however, we observed that *Cht6* isoform RG had a very similar expression pattern to the principal *dyl* isoform (also to overall *dyl* expression) (Fig 4B).

### Nature of the most highly expressed genes

We generated a spreadsheet that contained the 20 most highly expressed genes from each time point. Due to some genes being very highly expressed at multiple stages this was reduced to a set of 83 genes (S10 Table) that included genes with a wide variety of functions. For example, 9 of these encode known cytoskeleton proteins or effectors including *actin5C* and *alpha-Tubulin84B*. There were several enzyme encoding genes including *Pxd* [50], which encodes peroxidase and *Mmp1*, which encodes a metalloprotease [51]. Fourteen of the genes are annotated as cuticle protein genes in the fly genome and 2 others are homologs of genes annotated as cuticle protein genes in other insects (but not in Drosophila). Three additional genes encode proteins similar to ones identified as cuticle proteins by Mass Spec analysis of proteins extracted from Anopheles gambiae (Vectorbase). Eight of these 19 encode short proteins, less than 200 amino acids in length. In addition the amino acid content of many was unusual as is often seen in cuticle proteins [10, 15, 52]. Twelve lacked any Trp residues, 7 any Cys residues and there were also instances where proteins lacked any Arg, Asn, Asp, Gln or Glu. Four of the proteins contained greater than 20% Ala, 3 contained greater than 20% Val and there was one instance of greater than 20% Gln. An “average” protein does not possess more than 10% of any amino acid residue [53]. Three of the other most highly expressed genes (*dyl* [23, 24], *e* [45] and *pale* [44]) also play a role in cuticle formation. Thus, twenty two of the 83 genes are known to have a role in cuticle deposition. Among the remaining genes were 20 unstudied “CG” genes that are only found in arthropods. Interestingly, like the highly expressed annotated cuticle proteins 10 of the 20 encoded short (<200 aa) proteins and many also had a distinctive amino acid content. Nine did not contain any Trp, 5 did not contain any Asn, and 4 any Asp residues. There were also 3 instances of no Arg, 2 of no Cys, 2 of no Gln, 2 of no Glu, 2 of no Tyr, 1 of no His, 1 of no Phe and 1 of no Val residues. Two of the proteins contained greater than 20% Ala, 1 greater than 20% Val, 1 greater than 20% His and 1 greater than 30% Gly. We consider these genes candidates for encoding additional cuticle proteins or proteins that have specific functions in cuticle deposition. In addition, 7 members of the insect specific Osiris gene family [54] were among the 20 most highly expressed genes at 42 hr. The function of this family of genes is unknown. It is possible they function in cuticle formation or extracellular matrix secretion, assembly or modification.

### Changes in the expression of transcription factor encoding genes

We hypothesized that part of the mechanism responsible for the changes in gene expression we observed involved changes in the abundance of transcription factors. We queried FlyBase and obtained a list of 957 Drosophila genes annotated as having DNA binding activity. This was combined with a list of 384 genes annotated as having RNA PolII regulatory activity. Duplicates were removed leaving a list of 1077 genes and this was further reduced by only considering genes that had that had an FPKM of 30 or greater in at least one time point. Sixty six of these 385 genes had FPKM values that differed at least 4 fold between neighboring time points (see Fig 5D). We consider these genes to be good candidates for mediating the gene expression pattern for wing cuticle deposition. A number of genes were highly expressed in only one time point. For example, *HR4* was expressed at a much higher level at 52 hrs than at any of the other time points. Several genes showed elevated expression at more than one time point. For example, *aop* was highly expressed at 52 and 96 hr but not in the intervening time points.

### Isoform switching between subsequent time points

**Fig 6:**
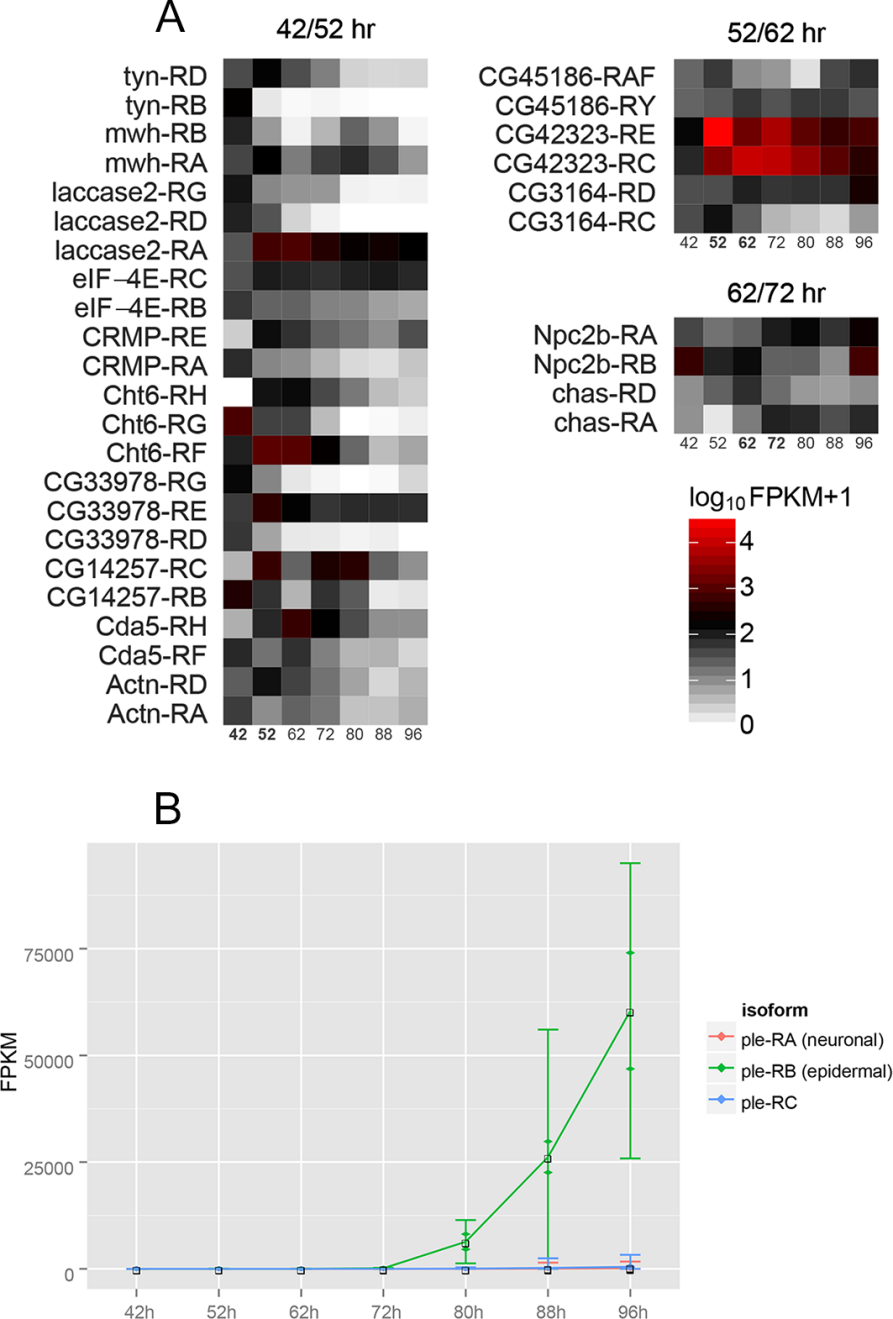
Isoform switching between neighboring time points. A. A heat map is shown for selected genes where we detected putative switching between isoforms. Several of these genes are known/likely to have a role in cuticle formation and or wing development. Among the most interesting were the isoform shifts for *Cht6* (which encodes a chitinase), *Cda5* (which encodes a chitin deacetylase), *laccase* (which encodes an oxidase with a role in cuticle formation [40, 57, 58]) and *mwh* (which regulates wing hair morphogenesis [55, 56]). B. The time course for the expression of the *pale* isoforms is shown. Note how the 96 hr peak of expression is restricted to the epidermal form.

We examined our data set for isoform changes and found 1362 cases of this (S11 Table). For many of these (664) the isoforms changed 10 fold or greater in abundance. As described earlier we confirmed the isoform switching of a pair of *Cht6* isoforms by RT-qPCR (Fig 4A). Having established that the relative FPKM values for individual isoforms are accurate across samples, we searched the dataset for isoform switching events between neighboring time points. The search returned a small number of genes from the early time points (42/52 hr: 10 genes, 23 isoforms; 52/62 hr: 3 genes, 6 isoforms; 62/72 hr: 2 genes, 4 isoforms), and none from the later ones (Fig 6A). Of particular interest was a pair of genes whose isoforms switched coordinately between 42 hr and 52 hr: *mwh* (*multiple wing hairs*) and *CG14257* (Fig 4C). *mwh* is a downstream component of the *frizzled/starry night* planar cell polarity pathway and mutations cause the development of multiple trichomes of abnormal polarity from individual wing epithelial cells [31, 55, 56]. Since the isoform FPKM values from RNA sequencing cannot be measured directly, but only inferred, we confirmed this result using RT-qPCR. These data show close co-expression of *mwh-RA* with *CG14257-RC*, and *mwh-RB* with *CG14257-RB* within the 42 hr—72 hr time frame (Fig 4C). Visual examination of Fig 6 suggests there are more pairs of genes expressed in this pattern.

The *pale* gene served as an internal control for the dataset. Its 3.7 kb transcript (ple-RA) is neuronal and the 3.2 kb transcript (ple-RB) is epidermal [59]. The vast majority of cells in the pupal wing are epidermal with only a relatively small number of neurons. *pale* was one of the most highly expressed genes in our dataset with a sharp peak at 96 hr awp. As expected our isoform analysis showed almost all of the expression was of the RB isoform (Fig 6B).

### Novel transcripts

Our analysis yielded one promising candidate for a new gene, annotated automatically as CUFF.2839 in locus: 3L:813,316.818,703 (3L:61D2). Three potential transcripts were found for this gene, one containing 2 exons and two containing 3 exons. Open reading frame and polypeptide searches did not yield any homologs. Another newly annotated sequence of interest was CUFF.3420.1, which contained an ORF whose corresponding polypeptide was in part identical to UniProtKB Q6ILG5—a theoretical unknown protein. Among all novel transcripts in unannotated regions, the only candidate we consider likely to be a true positive was CUFF.6.1, which encoded the TART transposon reverse transcriptase. Its locus was on an unplaced genome fragment 211000022280091. This transposon participates in the maintenance of Drosophila telomeres [60]. The results of the full search are presented S12 Table and the gene annotation file is provided in S2 File.

S12 Table contains unedited Cuffcompare (http://cole-trapnell-lab.github.io/cufflinks/cuffcompare/) output and complements the gene annotation file.

### Functional tests of “envelope candidate genes”

**Fig 7:**
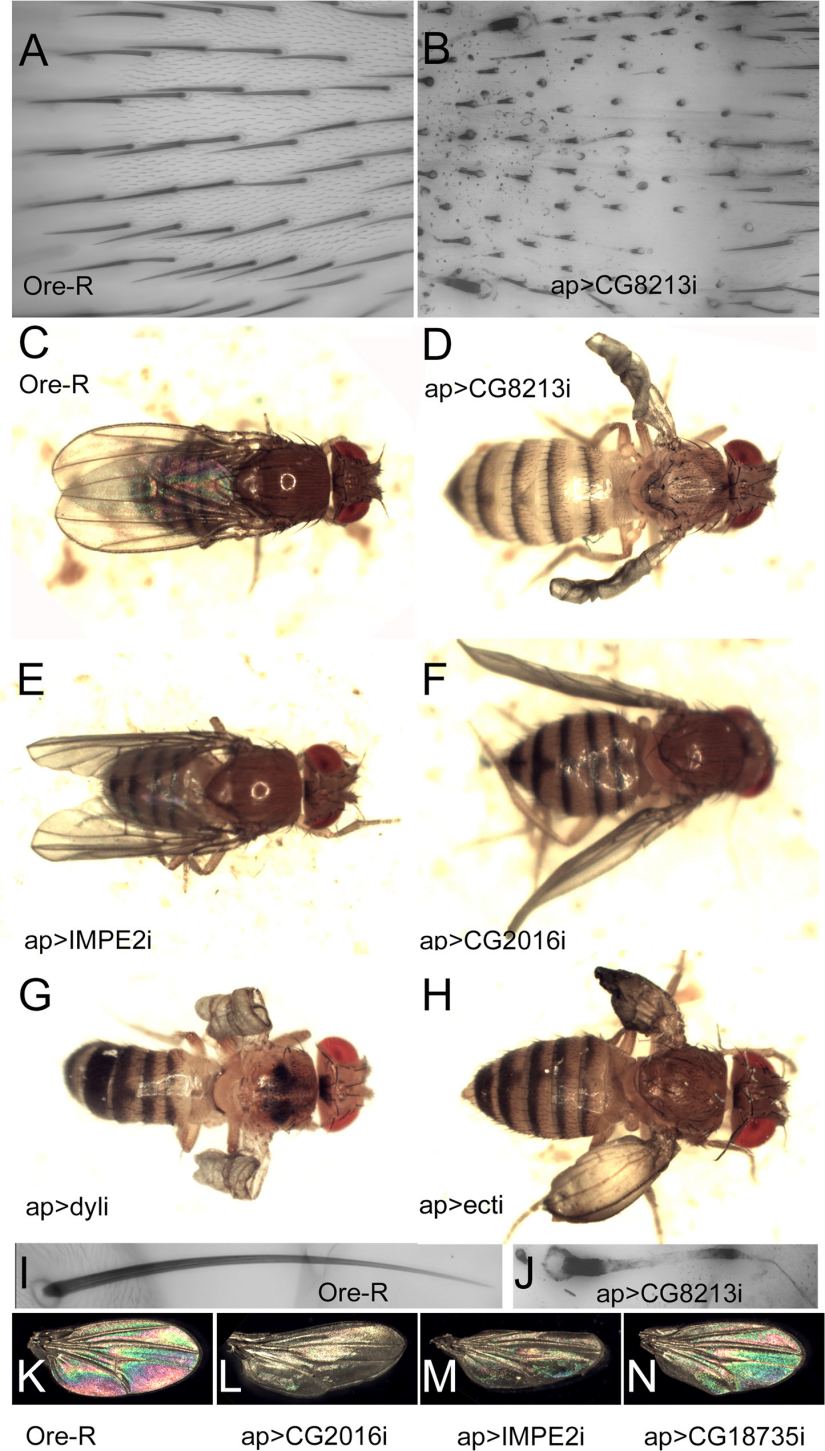
Phenotypes associated with 42 hr genes. A and B show the dorsal notum of Ore-R and *ap>CG8213*. *apterous-Gal4* drives expression in the cells that form the dorsal surface of the wing but not those that form the ventral surface. It also drives expression in the dorsal thorax (notum). Panels C-H show adult flies where a 42 hr gene was knocked down using *ap-Gal4*. Ore-R is shown (C) for comparison. I and J show a thoracic macrochaete from Ore-R and from *ap>CG8213*. Panels K-N show unmounted wings from Ore-R or knocked down 42 hr genes. Note the curved kd wings.

The literature establishes that mutations in several genes expressed almost exclusively at 42 hr (>90% of total FPKM values) result in gross defects in wing morphology. These include the *m* (93%), *dy* (96%) and *dyl* (93%) genes [18, 22]. For *m* and *dy* TEM examination showed that a loss of these two genes resulted in defects in cuticle structure that were obvious in the non-envelope cuticle layers [22]. For *dyl* we previously reported that chitin deposition was disrupted in hairs and bristles [18, 24], which would not be expected from an envelope defect. In the embryo mutations in a number of ZP domain genes including *m*, *dy* and *dyl* resulted in gross cuticle abnormalities in denticles [23]. We further characterized the *dyl* mutant phenotype and found that using *ap-Gal4* or *pnr-Gal4* to kd *dyl* led to dramatically abnormal cuticle pigmentation (S13 Table)(Fig 7G).

When *ap-Gal4* was used to knock down *dyl* in the dorsal but not ventral cell layer the adult wing curled upward (Fig 7G), consistent with the dorsal layer being smaller. There are several possible mechanisms that could give this result. It could be due to reduced proliferation or increased apoptosis in the dorsal layer. It could be due to a disruption of the flattening and expansion of the wing cells that starts around 42 hr, or it could be due to disruption of the unfolding and extension of the wing that happens right after eclosion (Fig 1 and S1 Fig). We did not see any gross abnormalities in wing discs or in pupal wings prior to flattening/expansion (Fig 1F, 1G, 1K, 1L, 1P and 1Q) thus the curled phenotype is unlikely to be due to effects on proliferation or apoptosis. In wild type after flattening/expansion the pupal wing has a reproducible folding pattern. This was altered in the *dyl* kd wings (Fig 1H–1J) indicating that *dyl* is required for normal wing flattening/expansion. This suggests that the envelope, which is being deposited at 42 hr, plays a functional role in this process. Since the *dyl* kd wings were abnormal at the time of eclosion we were not able to determine if the process of wing unfolding/extension could also be contributing to the *dyl* phenotype. We next examined *ap>dyl*-RNAi pupal wings in the TEM. The 44 hr pupal wings were not dramatically abnormal although we often observed hairs that looked abnormal on one wing surface. We note that equivalent TEM sections of *m* and *dy* pupal wings also did not appear highly abnormal [22]. Several types of abnormalities were observed in the older *dyl* kd pupal wings. In 76–80 hr apf wings there were large gaps between the epithelial cells and the cuticle (Fig 2H). This is reminiscent of what was observed in *dyl* mutant embryonic denticles [23]. It is worth noting that using *ap-Gal4* to drive the kd allows us to use the ventral wing cells as a control. Thus, we can be confident that abnormalities observed consistently in only one cell layer are not due to artifacts such as poor fixation as that would affect both cell layers. In addition to the large gaps we also consistently observed that the procuticle on the mutant side was significantly thicker (the ratio was 2.1 (sd = 0.43) compared to 1.2 for wt, p = 0.0017, t-test) (S1 Table), the procuticle appeared more variable with occasional “holes” and the adhesion layer appeared ragged. In some sections very abnormal procuticle was observed with large holes and irregularities (Fig 2K and 2L). The pupal hairs were also highly abnormal with an atypical shape and very thin cuticle (S3K Fig). Given how abnormal *dyl* mutant hairs are as observed by SEM, brightfield and confocal microscopy [18] this was not surprising.

We previously found that knocking down *ectodermal* (*ect*) (99% of total FPKM at 42 hr) function in the pupal wing led to wing hair defects [18]. When we kd *ect* using *ap-Gal4* or *pnr-Gal4* we observed notum abnormalities and frequently wings where the dorsal and ventral surfaces were not juxtaposed (i.e. wing blisters)(S13 Table and Fig 7H). The *ect* kd wings also often appeared smaller than normal. As was the case for the *dyl* kd wings, we did not observe defects in *ect* kd wing discs or pupal wings prior to flattening/expansion (Fig 1K and 1L), but such pupal wings showed a highly abnormal folding patterns after it (Fig 1M–1O). Thus, it appears that *ect* also functions in wing flattening/expansion. We examined *ap>ect*-RNAi pupal wings in the TEM and observed several defects (Fig 2I). As was the case for the *dyl* kd we also observed large gaps between the apical surface of the epithelial cells and the cuticle and the greater thickness of the dorsal vs ventral cuticle was enhanced (ratio = 1.79 (0.22)) compared to 1.2 for wt (p = 8 X10^-5^, t-test) (S1 Table). We did not, however, see the sort of holes in the procuticle seen with *dyl*. The *ect* pupal wing hairs also appeared abnormal as in proximal regions the hairs lacked the distinctive angular structure of wild type hairs (S2M Fig). This fits the phenotype seen in adult *ect* kd wings [18]. As was the case for the *dyl* kd we did not see any dramatic phenotype in the 42 hr wing as assayed by TEM.

We also chose 10 additional genes that were expressed primarily at 42 hr (>90% of total FPKM) and tested them to see if a kd would lead to wing and/or cuticle phenotypes. Seven gave a phenotype when kd with either *ptc-G4*, *ap-Gal4* or *pnr-Gal4* although in many cases these were weak in terms of expressivity or penetrance (S13 Table). Knocking down *CG8213* with *ap-Gal4* resulted in a severe curled upward wing phenotype as well as hair and notum bristle phenotypes (Fig 7D). The bristle phenotypes included very short bristles, bristles with wispy distal regions (Fig 7J) and bristles with varying regions of increased and decreased pigmentation (Fig 7J). This set of bristle phenotypes is not common but is also seen in knockdowns of *dyl*, *Rab11* and exocyst components [24]. *ap>CG2016*-RNAi resulted in wings with a distinctive upward curl (Fig 7F and 7L) and *ap>ImpE2*-RNAi wings with a distinctive downward curl (Fig 7E and 7M). These were not as strong as with CG8213 but they showed complete penetrance.

We investigated the *cg8213* kd phenotype further. As was the case for the *dyl* kd wings, we did not observe defects in wing discs or pupal wings prior to flattening/expansion (Fig 1P and 1Q), but abnormalities were obvious after (Fig 1R–1T). Thus, it appears that *CG8213* also functions in pupal wing flattening/expansion. We also examined CG8213 kd pupal wings by TEM. As was the case for *dyl* and *ect* we observed large gaps between the cuticle and the apical surface of the dorsal epithelial cells (Fig 2J). In contrast to the results with *dyl* and *ect* the cuticle on this side of the cell was also substantially thinner than that on the putative ventral side where the gaps were not seen. The V/D ratio was 2.29 (sd = 0.54) on average, significantly different from the putative V/D ratio of 0.82 in equivalent wild type wings (p = 2.9X10^-5^, t-test) (S1 Table). The difference was significant even if we assume the thicker layer in our wild type images was the ventral surface (p = 3.0 X 10^−5^). We also observed occasional regions where the procuticle lost its normal organized layered morphology (Fig 2M) and hairs with an abnormal shape (S2L Fig).

Since we did not carry out RNASeq on wings younger than 42 hr the data presented does not establish if the expression of the 42 hr genes actually peaked at 42 hr or if it could have peaked earlier and be declining at 42 hr. To assess this we made use of an earlier study from our lab of gene expression at 24, 32 and 40hr awp that used Affymetrix gene chips [61]. There were 48 “42 hr” genes with a total FPKM of over 100 and 45 of these were present in the chips used in our earlier study. Forty three of the 45 showed a higher level of expression at 40 hr than at 32 (or 24) hr (S14 Table). In most cases the increase in expression from 32 hr to 40 hr was quite large (median 12.4 fold increase). We conclude that the vast majority of 42 hr genes show a peak in expression around that time.

## Discussion

### Value of the data set for future studies on cuticle

Drosophila has served as a valuable model system for many molecular, cellular, developmental and behavioral problems. It has provided some important insights into cuticle synthesis, for example the role of ZP domain proteins in the process [22–24]. However, the number of such studies has remained limited and cuticle synthesis has remained poorly understood for a process so important for a large part of the biological world. The data set we describe here should provide a valuable resource for future studies on cuticle synthesis in Drosophila as well as other arthropods.

Many of the previous studies and reviews concerning insect cuticle focused on molting behavior. While understanding this process is important, it is complicated by the shedding of the old cuticle and the influence of the environment. The pupa is a closed system, which presumably reduces the variability of cuticle formation due to external conditions and stimuli. Wings, as single-tissue organs, are ideal for transcriptomic or proteomic studies on the development of cuticle. It remains to be established how variable the program for cuticle deposition will be from one body region to another.

### How many cuticle protein genes?

The Drosophila genome is annotated with almost 150 cuticle genes [15]. Our expectation is that cuticle genes would be expressed at a high level as structural proteins are typically needed in stoichiometric amounts. We also expected that homologs of these genes would be found in insects and perhaps other arthropods but not in the genomes of the many types of animals that do not produce a cuticular exoskeleton (e.g. vertebrates). Among the most highly expressed genes we identified 19 genes annotated as cuticle proteins either in the Drosophila genome or the genome of other insects. In this same set of very highly expressed genes were 20 unstudied genes that shared these properties. If the 20 unstudied genes prove to encode cuticle proteins it would more than double the number of highly expressed cuticle protein genes. Many annotated cuticle proteins are relatively short and many contain unusual amino acid content. Either or both of these properties were also true for many of these 20 genes consistent with the possibility that some may prove to be cuticle proteins.

The 20 genes considered above were selected because they were among the most highly expressed genes. Our data also hint that there may be many other genes expressed in the pupal wing that have specific roles in wing cuticle formation. Of the 16 gene expression clusters 10 displayed patterns of gene expression that changed dramatically in a stage specific manner and these clusters were significantly enriched for arthropod specific genes. This suggests that the number of genes with specific roles in cuticle formation could be very large.

### The cuticle deposition program

The metamorphic behavior of insects is under hormonal control and at least indirectly changes in ecdysone titer almost certainly influence the pattern of gene expression we describe during wing cuticle deposition. It is not clear however that the large number of changes we described are a direct read out of hormone levels. Ecdysone levels increase rapidly during the start of the second day awp formation peaking at 30 hrs. Two less dramatic peaks were seen at 40 and 48 hr with generally declining levels from 40 hr to eclosion [62]. It is not obvious that this pattern can explain our data. For example, ecdysone levels are similar at around 88 and 96 hr but we detect dramatic changes in gene expression. Further, based on simple observations of pupae we know that the time course of pupal development differs substantially across the body (e.g. abdominal development is delayed compared to thoracic development) but as far as we are aware there is no evidence for a similar spatial variation in ecdysone levels.

The literature provides validation that many genes we identified as being highly expressed in a dynamic fashion are essential for normal wing development and have phenotypes that implicate a role in cuticle formation or wing maturation. Several of the best examples are ZP domain proteins, which are known to organize apical extracellular matrix [23, 63]. In insects this matrix becomes the cuticle. Previous studies had found that ZP domain proteins played important roles in cuticle formation [18, 22–24]. In the embryo mutations in several ZP domain protein genes resulted in gaps between the apical surface of the epidermal cells and the cuticle [23], a result also seen previously for *m dy* double mutant wings [22] and one that we observed in pupae for *dyl* mutants. In our experiments this set of ZP domain genes were almost exclusively expressed in 42 hr pupal wings suggesting they functioned in envelope formation. In *dyl* kd and mutants we first observed a hair morphology phenotype in 39 hr wings [18]. This is during the deposition of the envelope but prior to when we first detect chitin in hairs and before epicuticle deposition, suggesting *dyl* functions in envelope deposition. We did not detect a clearly abnormal envelope in TEM images of 42 hr *dyl* kd wings but this could be due to thinness of the envelope as even in wild type it sometimes appears diffuse. At later stages gross abnormalities in procuticle were seen. In their study of *m* and *dy* wings Roch et al. [22] detected disorganization and a delay in formation of the extensions of the apical cytoplasm that are thought to represent sites of envelope cuticle deposition. This led them to suggest that M and Dy were either structural components of the envelope or linked the cuticle and epithelial cells. Some of the early literature suggested envelope formation was mediated by polymerization [64] which is the presumed function of *zona pellucida* domain. CG10005 stood out due to being highly expressed only in the 52 hr sample. It is a candidate for mediating some aspect of epicuticle formation; as an example of a protein with only the ZP-N domain present (most ZP-domain proteins contain ZP-N and ZP-C) it might be a good candidate for studying the function of ZPD proteins in subdomain detail.

The sclerotization of insect cuticle generally takes place very late in pupal development and shortly after eclosion. This process utilizes enzymes such as Tyrosine Hydroxylase, which is encoded by the *pale* gene [44] and Dopa Decarboxylase, which is encoded by the *Ddc* gene [46, 47]. Loss of function mutations in these genes lead to lightly pigmented, weak and fragile cuticle. Consistent with this biological function *pale* displayed a dramatic peak in expression at 96 hr awp, just prior to eclosion. There was a more than 12000-fold increase in *ple* expression between 42 and 96 hr awp and an almost 500-fold increase in the 24 hrs between 72 and 96 hr awp. The expression of Ddc did not change as dramatically but it showed a strong peak at 88 hr and 92 hr awp with a 27 fold increase from 62 to 96 hr. Thus, the expression pattern of these two key sclerotization genes nicely fit with their known biological function.

In our experiments the pigmentation of wing hairs was first obvious around 80 hr awp and it increased dramatically between then and 96 hr awp. There is a further increase during the first few hours of adult life. The expression of a number of genes known to be important for pigmentation varied during the period of cuticle deposition and provided further validation of our data set. Two of the most notable pigmentation genes are *ebony* (*e*) [45] and *black* (*b*) [65]. The expression of both of these showed a major peak at 96 hr awp. The *yellow* (*y*) gene is another key pigmentation gene and published data indicated it functioned in the middle of the pupal period [40]. Consistent with that *y* expression peaked at 52 hr awp in our experiments.

The genetic analysis of cuticle proteins in insects has been relatively limited, although in recent years a number of interesting studies have been published (e.g. [66–69]. In general these are based on examining the phenotype(s) associated with mutations that inactivate a single gene. It is important to distinguish between the mutant phenotype being a primary defect and not simply an end point that results from the lack of the gene product at an earlier execution point. The very sharp expression peaks that we found for some cuticle protein genes should facilitate such analyses.

Understanding of the genetic basis for the complex structure of insect cuticle remains challenging. We believe exclusivity of some genes to arthropods is a clue to narrow down the gene search space. A reasonable hypothesis is that the thickness of individual cuticular layers is dependent on the level of expression of a few major cuticle proteins and on the density of protein packing. Knowing the details of the gene expression program allows one to design tests of this hypothesis. A likely hypothesis to explain the change from one cuticle layer to another (e.g. envelope to epicuticle) is that a change in the abundance of one or a few transcription factors leads to a change in the array of cuticle proteins expressed. Our analysis suggests candidates that could mediate such changes. For example, the large reduction in the expression of *vri* and *CG14431* between 42 and 52 hrs and the steep increase in the expression of *HR4* and *Pdp1* at 52 hr could be responsible for the changes in gene expression that cause the transition from envelope to epicuticle.

The *shaven baby* (*svb/ovo*) transcription factor was not detected as having a high level of expression at 42 hr. At first glance this might be considered surprising as it is known to regulate the formation of larval denticles [70–72] and wing hairs [18, 73] and it functions to regulate the expression of a number of genes such as *dyl* and *m* whose expression was primarily found in 42 hr wings [14, 70, 72]. In previous experiments that utilized Affymetrix gene chips to examine pupal wing RNA at 24, 32 and 40 hrs awp we found a peak of *svb* RNA at 32 hr with a sharp decline by 40 hr [61]. Thus, our previous results are consistent with the current ones. We suggest that Svb is required to turn on the expression of genes such as *dyl* and *m* but either it is not required to maintain their expression or Svb is stable enough so that protein synthesized at 32 hr can drive a high level of target gene expression at 42 hr.

We analyzed a small collection of genes primarily expressed in 42 hr wings for their potential role in wing cuticle deposition. The results on CG8213 proved to be a particularly interesting. This gene encodes 3 large proteins (range 1430–1693 aa) each of which contains a serine protease domain [74]. A protein trap of CG8213 localized it to the perivitelline space in stage 11 and stage 15 embryos [75], indicating the protein is secreted, which would place it in a subcellular location to function in cuticle deposition. A perivitelline space localization was also seen for several other proteins likely to function in cuticle deposition (e.g Cda4 (Chitin deacetylase 4) and Dsx-c73A). The bristle phenotype observed with kd CG8213 was reminiscent of that seen with kd of *dyl* and genes such as *Rab11* that are needed for the secretion and/or plasma membrane insertion of Dyl [24]. This suggests the possibility that Dyl and CG8213 are functionally linked.

The timing for the formation of the cuticle that covers wing hairs appears to differ from that of the cuticle that covers the wing blade. This could be a complicating factor in the analysis of our data. We previously found that chitin could first be detected in 42 hr hairs while it not detected in the wing blade until later [18]. Thus, it is possible that some 42 hr genes might in fact function in the formation of internal hair cuticle layers and not envelope, although we suspect this would not be the case for the most highly expressed genes. Indeed, the formation of internal hair cuticle appears to be more rapid, and the epi- and procuticle are less easily distinguished than in the wing blade cuticle.

### The relationship of cuticle layers

**Fig 8:**
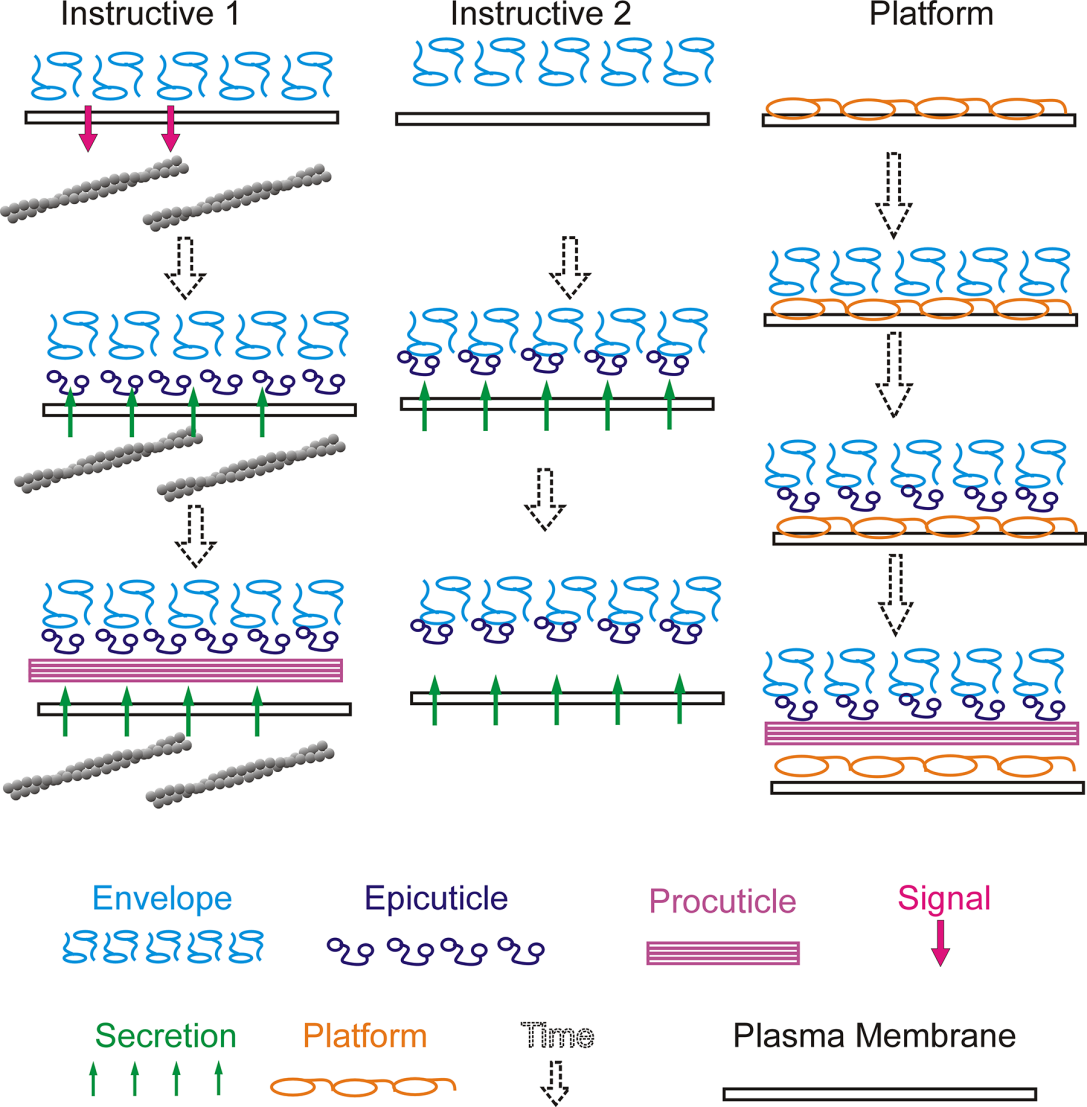
Models to explain the procuticle phenotypes of 42 hr genes. In the Instructive 1 model the 42 hr proteins form the envelope, which signals back to the cell to organize the cytoskeleton so that the epicuticle and procuticle is secreted in the proper pattern. In Instructive 2 the 42 hr proteins form the envelope and when the proteins that form the epicuticle are secreted they bind to and are patterned by envelope and 42 hr proteins. A similar situation could result in the epicuticle patterning the procuticle. In the Platform model the 42 hr proteins are not part of the envelope but they form a complex that is essential for the patterned secretion of the proteins that form the 3 cuticular layers. Our second model is that many of the 42 hr genes do not encode proteins that are part of the envelope. Rather they would form a “platform” or complex that in some way mediates the tight juxtaposition of the cuticle and the apical surface of the epithelial cells and that this platform is needed for the proper deposition of the envelope and other cuticular components [22].

There is usually a strict temporal relationship between the deposition of the various cuticle layers [37]. From the literature it is unclear if the deposition of the later layers is independent of or dependent on the earlier ones. If the layers were independent then loss of function mutations in genes that are only involved in envelope deposition would be expected to produce an altered envelope with normal epi- and procuticles. Since the envelope is such a small part of the cuticle thickness, envelope defects would only be expected to alter the surface of the cuticle and not its overall structure. Such defects might be visible as a rougher surface and it would not be surprising if they had functional effects on the hydrophobicity of the cuticle and/or made the animals more sensitive to desiccation. However, by combining the data in this paper and the literature it appears that mutations in genes expressed almost exclusively during envelope deposition often result in highly abnormal procuticle. We suggest two classes of models to explain this. In the first we hypothesize that the early layers are important for and perhaps instruct the deposition of the later ones. The first step in cuticle deposition—the formation of the envelope presumably involves proteins such as Dyl, Dy and M along with other candidates such as CG8213, Ect and Cpr76Bc. Dy and M have been hypothesized to be envelope components [22], an idea that we find attractive. Interactions between these proteins and cellular constituents such as apical transmembrane proteins and the cytoskeleton could provide the spatial and patterning information needed to build the envelope. Once the envelope is complete it would then guide the deposition of the epicuticle. This could be mediated by outside in signaling from the envelope to the epidermal cells with this leading to the epicuticle components being deposited in the correct spatial pattern (Fig 8). Support for such signaling comes from observations of abnormal apical cytoskeletons in *dyl*, *dy* and *m* mutants [18, 22, 24]. Alternatively, the patterning of secreted epicuticle components could be mediated by their binding directly to envelope components (Fig 8). It seems likely that this would only work for the deposition of the epicuticle that was at the envelope-epicuticle transition boundary. At a later stage interactions between the patterned boundary layer epicuticle proteins and newly secreted epicuticle proteins could pattern the remainder of the epicuticle. Equivalent interactions between the epicuticle and procuticle would serve to instruct the initial deposition of the procuticle. It is worth noting that the late maturation changes observed in the envelope and epicuticle show that cuticle deposition is not a strict temporal hierarchy. While most studies have argued for such a hierarchy a study on the deposition of the first instar larval cuticle of Drosophila came to a different conclusion [35].

The instructive model is appealing in that it does not require a structure yet to be identified (i.e. the platform) and it can explain the altered apical cytoskeleton seen in mutants. However, it does not provide an obvious explanation for the large gaps seen between the apical surface and the cuticle in mutants, although that could be due to defects in outside-in signaling. In contrast the “platform model” can explain the gaps, however it does not provide an explanation for the changes in the apical cytoskeleton. Given the multiplicity of cuticle-related proteins it may be that these models are too simple and that a model that contains aspects of both will be correct. It will be important to determine if the proteins in question are incorporated into the envelope as predicted by the instruction models.

The models suggested above assume that RNA levels are reflective of levels of synthesis of the relevant proteins. This is sometimes not precisely the case but in this system we find very large (often 100 fold or greater) changes in RNA levels so we doubt that changes in translational efficiency would be great enough to unlink RNA levels to general levels of protein synthesis. The model also assumes that the time of action of the relevant proteins is close to the time of their synthesis. Consistent with this are the cytoskeleton and other phenotypes seen in *dyl* mutants during the period of envelope deposition [18], as well as the sharp increase of *ple* expression with simultaneous cuticle darkening. We also note that the Dyl protein accumulated on the apical surface and/or apical extracellular matrix during envelope deposition consistent with deposition and secretion being closely linked temporally to the presence of RNA in pupal wing and bristle forming cells [18, 24].

### The apical extracellular matrix and cellular/tissue morphogenesis

The apical extracellular matrix is poorly understood compared to the basal extracellular matrix. Arthropod cuticle is not the only example of such a material as they are found in a wide variety of tissues, organs and organisms [76]. For example, the zona pellucida that surrounds mammalian oocytes [63] and the tectorial membrane of the cochlea [77, 78]. Studies on the secretion and morphogenesis of insect cuticle could provide insights into more general questions about how cells can organize their apical extracellular matrix and how it influences tissue function and development. Our observations establish that mutations in a number of “42 hr genes” disrupt the process of wing cell flattening and expansion. This could be due defects in outside in signaling regulating the cytoskeleton or to a proper physical connection between the apical surface of wing cells and the developing cuticle being required for mechanical regulation of morphogenesis. The importance of the apical extracellular matrix for morphogenesis is not unique to the wing. Extensive studies have found defects in trachea lumen morphogenesis associated with mutations in genes required for the deposition of the cuticle that covers the apical surface the trachea cells [20] [79] [80]. Connections between the pupal cuticle and imaginal disc cells are important for disc evagination and overall appendage morphology [81–83]. Apical extracellular matrix has also been found to be important for a number of other morphogenetic events including mediating the anchoring of sensory neurons to cuticle in Drosophila [84] and to anchor dendritic tips during cell body migration during the formation of the amphid sense organs in C. elegans [85]. Apical extracelluar matrix is also important for morphogenesis in early sea urchin embryo [86] and in mammals the ZP domain protein hensin is important for kidney collecting tube development [87] [88].

## Materials and Methods

### Collection and staging of animals

Oregon R flies were grown on standard fly food 25°C. White prepupae were collected and aged for varying lengths of time and pupal wings dissected in PBS (phosphate buffered saline). At the starting time point of our analysis (42 hr awp), the wings are rigid enough to minimize contamination by other tissues. A detailed protocol can be found in S1 File (Supplementary Methods). In a number of experiments we used *apterous-Gal4* (*ap-Gal4-* which drives expression in the cells that form the dorsal surface of the wing as well as the notum (dorsal thorax), *patched-Gal4* (*ptc-Gal4*, which drives expression in a stripe down the center of the wing and *pannier-Gal4* (*pnr-Gal4*, which drives expression in a stripe down the mid line of the notum and dorsal abdomen). The RNAi inducing transgenes used for knocking down gene expression contain the UAS sequence that is recognized by Gal4. Various lines came from either the VDRC or TRiP collections. The VDRC lines were obtained directly from the VDRC (http://stockcenter.vdrc.at/control/main) and the TRiP lines from the Bloomington Drosophila Stock Center (http://flystocks.bio.indiana.edu/). Those and other stocks obtained from the Bloomington Drosophila Stock Center (NIH P40OD018537) were used in this study.

### TEM

Wings were fixed in 2% glutaraldehyde, 4% paraformaldehyde, rinsed in 0.1 M pH 7.4 cacodylate buffer, post fixed with OsO_4_ and then rinsed again. After rinsing with distilled water the samples were dehydrated and then in SPURR resin. Sections were stained with 4% uranyl acetate and 0.25% lead citrate and examined in a JEOL 1230 transmission electron microscope. The published images were assembled using Adobe Photoshop.

To compare the thickness of the dorsal and ventral cuticle we used ImageJ to measure cuticle thickness (10 equally spaced measurements per sample point) from juxtaposed dorsal and ventral regions in low magnification TEM images (3-5K). A t-test was used to compare the average thicknesses for at least 6 such averaged measurements.

### RNA isolation and library construction

Details of the RNA isolation, manipulation and the RNASeq methods are provided in the S1 File (Supplemental Methods). Briefly, RNA was isolated from wings and replicate libraries made using NEBNextR Ultra RNA library Prep kit for IlluminaR. Multiplexed samples were run on an Illumina MiSeq machine.

### RT-qPCR confirmation of RNA-seq data

Primers specific to all or selected transcripts from single genes were designed using Primer-BLAST76 and Geneious (Biomatters Ltd.) and obtained from Eurofins MWG Operon. Primer-BLAST provides analysis of potential spurious targets. The RT-qPCR was done on an Applied Biosystems 7500 Fast Real Time PCR System. Error bars in the figures presenting RT-qPCR data are standard deviations of triplicate sample RQ as reported by system software. Libraries from the first biological replicate were used for RT-qPCR reactions. The calibrator sample was an equimolar mixture of each library from the first biological replicate. The RQ value is expression in each sample relative to the calibrator (which has the expression of 1). Details of primers used and methods can be found in the S1 and S3 Files (Supplementary Methods and MIQE data).

### Analysis of RNA-seq data

The adapter sequences needed for sequencing on the Illumina MiSeq platform were automatically trimmed on the BaseSpace website. The Tuxedo RNAseq analysis suite was used as described by the authors of the suite [89]. Between 79.5 and 89.4% of the reads in the 14 samples were successfully mapped to the Drosophila genome indicating that the sequence was of high quality. Cross-replicate variability was assessed by plotting squared coefficient of variation (CV^2^) against FPKM estimates for all quantified genes (S4 Fig). The variability tends to decrease with increasing FPKM. The 42 hr dataset is slightly the most variable one and 72, 80, 88 hr show the lowest CV^2^. Error bars for RNAseq data are 95% confidence intervals as reported by Cuffdiff [29] [90]. Downstream analysis of differential expression results, including production of heat map graphs, was done in R with the help of package cummeRbund and its dependencies. Gene clustering was done using a k-means algorithm (partitioning around medoids). Other aspects of the analysis of differential expression were done in Excel. The reads were mapped to FlyBase genome version 6.02, which contains 17234 genes and 33634 isoforms. Sequencing and mapping data are available for download from NCBI (BioProject accession code: PRJNA259920). For Fig 4, the theoretical FPKM for each gene in the calibrator sample was calculated as an arithmetic mean of FPKM values in each sample. A more detailed description is provided in the S1 File.

## Supporting Information

S1 Fig Changes in pupal wing morphology over time. All images are light micrographs shown at the same magnification. Panel A– 42 hr pupal wing, B– 62 hr pupal wing, C– 80 hr pupal wing, D– 96 hr pupal wing and E an adult wing. For panels A and B the field diaphragm was partially shut to increase contrast. The time is for pupae collected as white prepupae and aged at 25°C. Note the pigmentation that becomes obvious at 80 hr. When relatively young wings are dissected they “relax” so that they are not as tightly folded back as they appeared in the pupae.
(TIF)

S2 Fig Transmission electron microscopy of 38 hr pupal wings. A shows the secretion of relatively electron dense material (arrowheads) from putative undulae in the region of future wing blade. B and C are cross sections of developing hairs. The arrows point to patches of typical envelope and the arrowheads to more amorphous electron dense material. B shows a number of patches of envelope while C does not. All of these images came from the same wing.
(TIF)

S3 Fig Transmission Electron Microscopy of wing hairs and cuticle deposition. A. In 42 hr awp wings there are interruptions in the envelope (arrowhead) and cytoplasmic protrusions are seen where there is close contact between the envelope and the cytoplasm (arrows). The protrusions are thought to be sites of cuticle deposition B. Cytoplasmic protrusions (arrows) that are closely juxtaposed to the cuticle are also seen in later wings (72 hr awp). Both A and B at shown at the same magnification. Panels C-F and I are cross sections through developing hairs at progressively later stages. All of these images are shown at the same magnification. In panel C (42 hr) the developing envelope is incomplete with gaps present (arrowhead). By 52 hr awp (D) the hairs have taken on their fluted shape. Dark areas are prominent in the 88 and 96 hr awp hairs (arrow) (FI) that likely represent pigment deposition. G and H show glancing sections that show developing hairs present on pedestals (arrows). Note the hair pigmentation spreads into the pedestals. J-M are hairs from 76–80 hr wings where a 42 hr gene was kd using *ap-Gal4*. J is a control hair from the ventral surface of the same wing as K (dorsal surface of *ap>dyl-RNAi)*.
(TIF)

S4 Fig Cross Replicate Variability of RNAseq sequencing runs. Plotted is the squared coefficient of variation (CV^2^) for genes with an expression level of FPKM≥10 as a function of log_10_FPKM. Note that the CV^2^ tended to decrease with increasing expression level and that the 42 hr sample showed the most variability. That sample was the most difficult to dissect and hence has the greatest chance of contamination by other cell types.
(PDF)

S1 Table The dorsal/ventral cuticle thickness is provided for wild type and mutant wings. (PDF)

S2 Table FPKM values for all genes at all time points. (XLSX)

S3 Table A comparison of the number of genes whose expression changed between neighboring time points. (PDF)

S4 Table Comparison of expression levels for all genes at all pairs of time points. (XLSX)

S5 Table The names of each gene in each gene cluster. The medoid is in bold.
(XLSX)

S6 Table The number of genes whose expression level (FPKM) at any time point is greater than 90% (80%, 50%) of the sum of expression at all time points. (PDF)

S7 Table Different gene expression clusters show different frequencies of mutant phenotypes and restriction of genes t arthropods. (PDF)

S8 Table Gene ontology analysis of gene expression clusters. (PDF)

S9 Table Expression of cuticle protein encoding genes during wing cuticle deposition. (XLSX)

S10 Table Properties of the 20 most highly expressed genes at each time point. (XLSX)

S11 Table Expression of gene isoforms at the different time points. (XLS)

S12 Table Search for novel genes from the RNA Seq data. (XLSX)

S13 Table Phenotypes associated with knockdowns of “42 hr genes”. (XLSX)

S14 Table The vast majority of “42 hr genes” were previously found to be expressed at a higher level in 40 hr vs 32 hr pupal wings. (XLSX)

S1 File Supplementary methods. (DOCX)

S2 File Genome annotation file. (GTF)

S3 File MIQE archive. (ZIP)

## References

[1] Vincent JF, Wegst UG. Design and mechanical properties of insect cuticle. Arthropod structure & development. 2004;33(3):187–99. 18089034 10.1016/j.asd.2004.05.006

[2] Moussian B, Veerkamp J, Muller U, Schwarz H. Assembly of the Drosophila larval exoskeleton requires controlled secretion and shaping of the apical plasma membrane. Matrix biology: journal of the International Society for Matrix Biology. 2007;26(5):337–47. 17360167 10.1016/j.matbio.2007.02.001

[3] Payre F. Genetic control of epidermis differentiation in Drosophila. The International journal of developmental biology. 2004;48(2–3):207–15. 15272387

[4] Vincent JF. Deconstructing the design of a biological material. Journal of theoretical biology. 2005;236(1):73–8. 15967184 10.1016/j.jtbi.2005.02.017

[5] Grunenfelder LK, Herrera S, Kisailus D. Crustacean-derived biomimetic components and nanostructured composites. Small. 2014;10(16):3207–32. 10.1002/smll.201400559 24833136

[6] Rajabi H, Darvizeh A, Shafiei A, Taylor D, Dirks JH. Numerical investigation of insect wing fracture behaviour. Journal of biomechanics. 2015;48(1):89–94. 10.1016/j.jbiomech.2014.10.037 25468669

[7] Hojrup P, Andersen SO, Roepstorff P. Isolation, characterization, and N-terminal sequence studies of cuticular proteins from the migratory locust, Locusta migratoria. European journal of biochemistry / FEBS. 1986;154(1):153–9. 3943519 10.1111/j.1432-1033.1986.tb09371.x

[8] Suderman RJ, Andersen SO, Hopkins TL, Kanost MR, Kramer KJ. Characterization and cDNA cloning of three major proteins from pharate pupal cuticle of Manduca sexta. Insect biochemistry and molecular biology. 2003;33(3):331–43. 12609518 10.1016/s0965-1748(02)00247-3

[9] He N, Botelho JM, McNall RJ, Belozerov V, Dunn WA, Mize T, et al Proteomic analysis of cast cuticles from Anopheles gambiae by tandem mass spectrometry. Insect biochemistry and molecular biology. 2007;37(2):135–46. 17244542 10.1016/j.ibmb.2006.10.011

[10] Cornman RS. The distribution of GYR- and YLP-like motifs in Drosophila suggests a general role in cuticle assembly and other protein-protein interactions. PloS one. 2010;5(9). 10.1371/journal.pone.0012536 PMC2932725 20824096

[11] Snyder M, Hunkapiller M, Yuen D, Silvert D, Fristrom J, Davidson N. Cuticle protein genes of Drosophila: structure, organization and evolution of four clustered genes. Cell. 1982;29(3):1027–40. 6817923 10.1016/0092-8674(82)90466-4

[12] Karouzou MV, Spyropoulos Y, Iconomidou VA, Cornman RS, Hamodrakas SJ, Willis JH. Drosophila cuticular proteins with the R&R Consensus: annotation and classification with a new tool for discriminating RR-1 and RR-2 sequences. Insect biochemistry and molecular biology. 2007;37(8):754–60. 17628275 10.1016/j.ibmb.2007.03.007

[13] Ioannidou ZS, Theodoropoulou MC, Papandreou NC, Willis JH, Hamodrakas SJ. CutProtFam-Pred: detection and classification of putative structural cuticular proteins from sequence alone, based on profile hidden Markov models. Insect biochemistry and molecular biology. 2014;52:51–9. 10.1016/j.ibmb.2014.06.004 24978609 PMC4143468

[14] Andrew DJ, Baker BS. Expression of the Drosophila secreted cuticle protein 73 (dsc73) requires Shavenbaby. Developmental dynamics: an official publication of the American Association of Anatomists. 2008;237(4):1198–206. 18351665 10.1002/dvdy.21512 PMC2826221

[15] Willis JH. Structural cuticular proteins from arthropods: annotation, nomenclature, and sequence characteristics in the genomics era. Insect biochemistry and molecular biology. 2010;40(3):189–204. 10.1016/j.ibmb.2010.02.001 20171281 PMC2872936

[16] Gagou ME, Kapsetaki M, Turberg A, Kafetzopoulos D. Stage-specific expression of the chitin synthase DmeChSA and DmeChSB genes during the onset of Drosophila metamorphosis. Insect biochemistry and molecular biology. 2002;32(2):141–6. 11755055 10.1016/s0965-1748(01)00101-1

[17] Moussian B, Schwarz H, Bartoszewski S, Nusslein-Volhard C. Involvement of chitin in exoskeleton morphogenesis in Drosophila melanogaster. Journal of morphology. 2005;264(1):117–30. 15747378 10.1002/jmor.10324

[18] Adler PN, Sobala LF, Thom D, Nagaraj R. dusky-like is required to maintain the integrity and planar cell polarity of hairs during the development of the Drosophila wing. Developmental biology. 2013;379(1):76–91. 10.1016/j.ydbio.2013.04.012 23623898 PMC3686509

[19] Xi Y, Pan PL, Ye YX, Yu B, Xu HJ, Zhang CX. Chitinase-like gene family in the brown planthopper, Nilaparvata lugens. Insect molecular biology. 2015;24(1):29–40. 10.1111/imb.12133 25224926

[20] Moussian B, Tang E, Tonning A, Helms S, Schwarz H, Nusslein-Volhard C, et al Drosophila Knickkopf and Retroactive are needed for epithelial tube growth and cuticle differentiation through their specific requirement for chitin filament organization. Development. 2006;133(1):163–71. 16339194 10.1242/dev.02177

[21] Chaudhari SS, Arakane Y, Specht CA, Moussian B, Boyle DL, Park Y, et al Knickkopf protein protects and organizes chitin in the newly synthesized insect exoskeleton. Proceedings of the National Academy of Sciences of the United States of America. 2011;108(41):17028–33. 10.1073/pnas.1112288108 21930896 PMC3193238

[22] Roch F, Alonso CR, Akam M. Drosophila miniature and dusky encode ZP proteins required for cytoskeletal reorganisation during wing morphogenesis. Journal of cell science. 2003;116(Pt 7):1199–207. 10.1242/jcs.00298 12615963

[23] Fernandes I, Chanut-Delalande H, Ferrer P, Latapie Y, Waltzer L, Affolter M, et al Zona pellucida domain proteins remodel the apical compartment for localized cell shape changes. Developmental cell. 2010;18(1):64–76. 10.1016/j.devcel.2009.11.009 20152178

[24] Nagaraj R, Adler PN. Dusky-like functions as a Rab11 effector for the deposition of cuticle during Drosophila bristle development. Development. 2012;139(5):906–16. 10.1242/dev.074252 22278919 PMC3274354

[25] Arakane Y, Muthukrishnan S, Beeman RW, Kanost MR, Kramer KJ. Laccase 2 is the phenoloxidase gene required for beetle cuticle tanning. Proceedings of the National Academy of Sciences of the United States of America. 2005;102(32):11337–42. 16076951 10.1073/pnas.0504982102 PMC1183588

[26] Arakane Y, Lomakin J, Beeman RW, Muthukrishnan S, Gehrke SH, Kanost MR, et al Molecular and functional analyses of amino acid decarboxylases involved in cuticle tanning in Tribolium castaneum. The Journal of biological chemistry. 2009;284(24):16584–94. 10.1074/jbc.M901629200 19366687 PMC2713571

[27] Suderman RJ, Dittmer NT, Kanost MR, Kramer KJ. Model reactions for insect cuticle sclerotization: cross-linking of recombinant cuticular proteins upon their laccase-catalyzed oxidative conjugation with catechols. Insect biochemistry and molecular biology. 2006;36(4):353–65. 16551549 10.1016/j.ibmb.2006.01.012

[28] Cloonan N, Grimmond SM. Transcriptome content and dynamics at single-nucleotide resolution. Genome biology. 2008;9(9):234 10.1186/gb-2008-9-9-234 18828881 PMC2592708

[29] Trapnell C, Hendrickson DG, Sauvageau M, Goff L, Rinn JL, Pachter L. Differential analysis of gene regulation at transcript resolution with RNA-seq. Nature biotechnology. 2013;31(1):46–53. 10.1038/nbt.2450 23222703 PMC3869392

[30] Fristrom D, Wilcox M, Fristrom J. The distribution of PS integrins, laminin A and F-actin during key stages in Drosophila wing development. Development. 1993;117(2):509–23. 8330522 10.1242/dev.117.2.509

[31] Wong LL, Adler PN. Tissue polarity genes of Drosophila regulate the subcellular location for prehair initiation in pupal wing cells. The Journal of cell biology. 1993;123(1):209–21. 8408199 10.1083/jcb.123.1.209 PMC2119819

[32] Turner CM, Adler PN. Distinct roles for the actin and microtubule cytoskeletons in the morphogenesis of epidermal hairs during wing development in Drosophila. Mechanisms of development. 1998;70(1–2):181–92. 9510034 10.1016/s0925-4773(97)00194-9

[33] Guild GM, Connelly PS, Ruggiero L, Vranich KA, Tilney LG. Actin filament bundles in Drosophila wing hairs: hairs and bristles use different strategies for assembly. Molecular biology of the cell. 2005;16(8):3620–31. 15917291 10.1091/mbc.E05-03-0185 PMC1182302

[34] Sobala LF, Wang Y, Adler PN. ChtVis-Tomato, a genetic reporter for in vivo visualization of chitin deposition in Drosophila. Development. 2015;142:in press. 10.1242/dev.126987 PMC4712883 26395478

[35] Moussian B, Seifarth C, Muller U, Berger J, Schwarz H. Cuticle differentiation during Drosophila embryogenesis. Arthropod structure & development. 2006;35(3):137–52. 18089066 10.1016/j.asd.2006.05.003

[36] Shaik KS, Meyer F, Vazquez AV, Flotenmeyer M, Cerdan ME, Moussian B. delta-Aminolevulinate synthase is required for apical transcellular barrier formation in the skin of the Drosophila larva. European journal of cell biology. 2012;91(3):204–15. 10.1016/j.ejcb.2011.11.005 22293958

[37] Locke M. Pore canals and realted structures in insect cuticle. The journal of Biophysics and Biochemical Cytology. 1961;10:589–618. 10.1083/jcb.10.4.589 PMC2225106 13762980

[38] Schmidt EL. Observations on the subcuticular layer in the insect integument. J Morph,. 1956;99:211

[39] Mitchell HK, Edens J, Petersen NS. Stages of cell hair construction in Drosophila. Developmental genetics. 1990;11(2):133–40. 2116250 10.1002/dvg.1020110203

[40] Riedel F, Vorkel D, Eaton S. Megalin-dependent yellow endocytosis restricts melanization in the Drosophila cuticle. Development. 2011;138(1):149–58. 10.1242/dev.056309 21138977

[41] Mummery-Widmer JL, Yamazaki M, Stoeger T, Novatchkova M, Bhalerao S, Chen D, et al Genome-wide analysis of Notch signalling in Drosophila by transgenic RNAi. Nature. 2009;458(7241):987–92. 10.1038/nature07936 19363474 PMC2988197

[42] Shahin R, Iwanaga M, Kawsaki H. Cuticular protein and transcription factor genes expressed during prepupal–pupal transition and by ecdysone pulse treatment in wing discs of Bombyx mori. Insect molecular biology. 2016:in press. 10.1111/imb.12207 26748620

[43] Ostrowski S, Dierick HA, Bejsovec A. Genetic control of cuticle formation during embryonic development of Drosophila melanogaster. Genetics. 2002;161(1):171–82. 12019232 10.1093/genetics/161.1.171 PMC1462094

[44] Neckameyer WS, Quinn WG. Isolation and characterization of the gene for Drosophila tyrosine hydroxylase. Neuron. 1989;2(2):1167–75. 2483109 10.1016/0896-6273(89)90183-9

[45] Hovemann BT, Ryseck RP, Walldorf U, Stortkuhl KF, Dietzel ID, Dessen E. The Drosophila ebony gene is closely related to microbial peptide synthetases and shows specific cuticle and nervous system expression. Gene. 1998;221(1):1–9. 9852943 10.1016/s0378-1119(98)00440-5

[46] Hirsh J, Davidson N. Isolation and characterization of the dopa decarboxylase gene of Drosophila melanogaster. Molecular and cellular biology. 1981;1(6):475–85. 6086012 10.1128/mcb.1.6.475-485.1981 PMC369691

[47] Wright TR, Hodgetts RB, Sherald AF. The genetics of dopa decarboxylase in Drosophila melanogaster. I. Isolation and characterization of deficiencies that delete the dopa-decarboxylase-dosage-sensitive region and the alpha-methyl-dopa-hypersensitive locus. Genetics. 1976;84(2):267–85. 826447 10.1093/genetics/84.2.267 PMC1213576

[48] Biessmann H. Molecular analysis of the yellow gene (y) region of Drosophila melanogaster. Proceedings of the National Academy of Sciences of the United States of America. 1985;82(21):7369–73. 3933004 10.1073/pnas.82.21.7369 PMC391346

[49] Geyer PK, Spana C, Corces VG. On the molecular mechanism of gypsy-induced mutations at the yellow locus of Drosophila melanogaster. The EMBO journal. 1986;5(10):2657–62. 3096713 10.1002/j.1460-2075.1986.tb04548.x PMC1167166

[50] Konstandi OA, Papassideri IS, Stravopodis DJ, Kenoutis CA, Hasan Z, Katsorchis T, et al The enzymatic component of Drosophila melanogaster chorion is the Pxd peroxidase. Insect biochemistry and molecular biology. 2005;35(9):1043–57. 15979004 10.1016/j.ibmb.2005.04.005

[51] Llano E, Pendas AM, Aza-Blanc P, Kornberg TB, Lopez-Otin C. Dm1-MMP, a matrix metalloproteinase from Drosophila with a potential role in extracellular matrix remodeling during neural development. The Journal of biological chemistry. 2000;275(46):35978–85. 10964925 10.1074/jbc.M006045200

[52] Cornman RS, Willis JH. Annotation and analysis of low-complexity protein families of Anopheles gambiae that are associated with cuticle. Insect molecular biology. 2009;18(5):607–22. 10.1111/j.1365-2583.2009.00902.x 19754739 PMC3701952

[53] Trinquier G, Sanejouand YH. Which effective property of amino acids is best preserved by the genetic code? Protein Eng. 1998;11(3):153–69. 9613840 10.1093/protein/11.3.153

[54] Dorer DR, Rudnick JA, Moriyama EN, Christensen AC. A family of genes clustered at the Triplo-lethal locus of Drosophila melanogaster has an unusual evolutionary history and significant synteny with Anopheles gambiae. Genetics. 2003;165(2):613–21. 14573474 10.1093/genetics/165.2.613 PMC1462804

[55] Strutt D, Warrington SJ. Planar polarity genes in the Drosophila wing regulate the localisation of the FH3-domain protein Multiple Wing Hairs to control the site of hair production. Development. 2008;135(18):3103–11. 10.1242/dev.025205 18701542 PMC2556872

[56] Yan J, Huen D, Morely T, Johnson G, Gubb D, Roote J, et al The multiple-wing-hairs Gene Encodes a Novel GBD-FH3 Domain-Containing Protein That Functions Both Prior to and After Wing Hair Initiation. Genetics. 2008;180(1):219–28. 10.1534/genetics.108.091314 18723886 PMC2535676

[57] Sugumaran M, Giglio L, Kundzicz H, Saul S, Semensi V. Studies on the enzymes involved in puparial cuticle sclerotization in Drosophila melanogaster. Archives of insect biochemistry and physiology. 1992;19(4):271–83. 1600191 10.1002/arch.940190406

[58] Jacobs CG, Braak N, Lamers GE, van der Zee M. Elucidation of the serosal cuticle machinery in the beetle Tribolium by RNA sequencing and functional analysis of Knickkopf1, Retroactive and Laccase2. Insect biochemistry and molecular biology. 2015;60:7–12. 10.1016/j.ibmb.2015.02.014 25747006

[59] Birman S, Morgan B, Anzivino M, Hirsh J. A novel and major isoform of tyrosine hydroxylase in Drosophila is generated by alternative RNA processing. The Journal of biological chemistry. 1994;269(42):26559–67. 7929381

[60] Sheen FM, Levis RW. Transposition of the LINE-like retrotransposon TART to Drosophila chromosome termini. Proceedings of the National Academy of Sciences of the United States of America. 1994;91(26):12510–4. 7809068 10.1073/pnas.91.26.12510 PMC45468

[61] Ren N, Zhu C, Lee H, Adler PN. Gene expression during Drosophila wing morphogenesis and differentiation. Genetics. 2005;171(2):625–38. 15998724 10.1534/genetics.105.043687 PMC1456776

[62] Handler AM. Ecdysteroid titers during pupal and adult development in Drosophila melanogaster. Developmental biology. 1982;93(1):73–82. 6813165 10.1016/0012-1606(82)90240-8

[63] Jovine L, Darie CC, Litscher ES, Wassarman PM. Zona pellucida domain proteins. Annual review of biochemistry. 2005;74:83–114. 15952882 10.1146/annurev.biochem.74.082803.133039

[64] Wigglesworth VB. The Principles of Insect Physiology. London, UK: Chapman and Hll; 1972.

[65] Ashburner M, Misra S, Roote J, Lewis SE, Blazej R, Davis T, et al An exploration of the sequence of a 2.9-Mb region of the genome of Drosophila melanogaster: the Adh region. Genetics. 1999;153(1):179–219. 10471707 10.1093/genetics/153.1.179 PMC1460734

[66] Shaik KS, Wang Y, Aravind L, Moussian B. The Knickkopf DOMON domain is essential for cuticle differentiation in Drosophila melanogaster. Archives of insect biochemistry and physiology. 2014;86(2):100–6. 10.1002/arch.21165 24723222

[67] Qiao L, Xiong G, Wang RX, He SZ, Chen J, Tong XL, et al Mutation of a cuticular protein, BmorCPR2, alters larval body shape and adaptability in silkworm, Bombyx mori. Genetics. 2014;196(4):1103–15. 10.1534/genetics.113.158766 24514903 PMC3982684

[68] Noh MY, Kramer KJ, Muthukrishnan S, Kanost MR, Beeman RW, Arakane Y. Two major cuticular proteins are required for assembly of horizontal laminae and vertical pore canals in rigid cuticle of Tribolium castaneum. Insect biochemistry and molecular biology. 2014;53:22–9. 10.1016/j.ibmb.2014.07.005 25042128

[69] Chaudhari SS, Arakane Y, Specht CA, Moussian B, Kramer KJ, Muthukrishnan S, et al Retroactive maintains cuticle integrity by promoting the trafficking of Knickkopf into the procuticle of Tribolium castaneum. PLoS genetics. 2013;9(1):e1003268 10.1371/journal.pgen.1003268 23382702 PMC3561106

[70] Chanut-Delalande H, Fernandes I, Roch F, Payre F, Plaza S. Shavenbaby couples patterning to epidermal cell shape control. PLoS biology. 2006;4(9):e290 16933974 10.1371/journal.pbio.0040290 PMC1551925

[71] Kondo T, Plaza S, Zanet J, Benrabah E, Valenti P, Hashimoto Y, et al Small peptides switch the transcriptional activity of Shavenbaby during Drosophila embryogenesis. Science. 2010;329(5989):336–9. 10.1126/science.1188158 20647469

[72] Menoret D, Santolini M, Fernandes I, Spokony R, Zanet J, Gonzalez I, et al Genome-wide analyses of Shavenbaby target genes reveals distinct features of enhancer organization. Genome biology. 2013;14(8):R86 10.1186/gb-2013-14-8-r86 23972280 PMC4053989

[73] Delon I, Chanut-Delalande H, Payre F. The Ovo/Shavenbaby transcription factor specifies actin remodelling during epidermal differentiation in Drosophila. Mechanisms of development. 2003;120(7):747–58. 12915226 10.1016/s0925-4773(03)00081-9

[74] Dos Santos G, Schroeder AJ, Goodman JL, Strelets VB, Crosby MA, Thurmond J, et al FlyBase: introduction of the Drosophila melanogaster Release 6 reference genome assembly and large-scale migration of genome annotations. Nucleic acids research. 2015;43(Database issue):D690–7. 10.1093/nar/gku1099 25398896 PMC4383921

[75] Lye CM, Naylor HW, Sanson B. Subcellular localisations of the CPTI collection of YFP-tagged proteins in Drosophila embryos. Development. 2014;141(20):4006–17. 10.1242/dev.111310 25294944 PMC4197698

[76] Brown NH. Extracellular matrix in development: insights from mechanisms conserved between invertebrates and vertebrates. Cold Spring Harb Perspect Biol. 2011;3(12). 10.1101/cshperspect.a005082 PMC3225944 21917993

[77] Richardson GP, Lukashkin AN, Russell IJ. The tectorial membrane: one slice of a complex cochlear sandwich. Curr Opin Otolaryngol Head Neck Surg. 2008;16(5):458–64. 10.1097/MOO.0b013e32830e20c4 18797289 PMC2874155

[78] Sagong B, Park R, Kim YH, Lee KY, Baek JI, Cho HJ, et al Two novel missense mutations in the TECTA gene in Korean families with autosomal dominant nonsyndromic hearing loss. Ann Clin Lab Sci. 2010;40(4):380–5. 20947814

[79] Araujo SJ, Aslam H, Tear G, Casanova J. mummy/cystic encodes an enzyme required for chitin and glycan synthesis, involved in trachea, embryonic cuticle and CNS development—analysis of its role in Drosophila tracheal morphogenesis. Developmental biology. 2005;288(1):179–93. 16277981 10.1016/j.ydbio.2005.09.031

[80] Tonning A, Hemphala J, Tang E, Nannmark U, Samakovlis C, Uv A. A transient luminal chitinous matrix is required to model epithelial tube diameter in the Drosophila trachea. Developmental cell. 2005;9(3):423–30. 16139230 10.1016/j.devcel.2005.07.012

[81] Turner CM, Adler PN. Morphogenesis of Drosophila pupal wings in vitro. Mechanisms of development. 1995;52(2–3):247–55. 8541213 10.1016/0925-4773(95)00405-p

[82] Ray RP, Matamoro-Vidal A, Ribeiro PS, Tapon N, Houle D, Salazar-Ciudad I, et al Patterned Anchorage to the Apical Extracellular Matrix Defines Tissue Shape in the Developing Appendages of Drosophila. Developmental cell. 2015;34(3):310–22. 10.1016/j.devcel.2015.06.019 26190146 PMC4539345

[83] Etournay R, Popovic M, Merkel M, Nandi A, Blasse C, Aigouy B, et al Interplay of cell dynamics and epithelial tension during morphogenesis of the Drosophila pupal wing. eLife. 2015;4:e07090 10.7554/eLife.07090 26102528 PMC4574473

[84] Chung YD, Zhu J, Han Y, Kernan MJ. nompA encodes a PNS-specific, ZP domain protein required to connect mechanosensory dendrites to sensory structures. Neuron. 2001;29(2):415–28. 11239432 10.1016/s0896-6273(01)00215-x

[85] Heiman MG, Shaham S. DEX-1 and DYF-7 establish sensory dendrite length by anchoring dendritic tips during cell migration. Cell. 2009;137(2):344–55. 10.1016/j.cell.2009.01.057 19344940 PMC2673108

[86] Lane MC, Koehl MA, Wilt F, Keller R. A role for regulated secretion of apical extracellular matrix during epithelial invagination in the sea urchin. Development. 1993;117(3):1049–60. 8325234 10.1242/dev.117.3.1049

[87] Gao X, Eladari D, Leviel F, Tew BY, Miro-Julia C, Cheema FH, et al Deletion of hensin/DMBT1 blocks conversion of beta- to alpha-intercalated cells and induces distal renal tubular acidosis. Proceedings of the National Academy of Sciences of the United States of America. 2010;107(50):21872–7. 10.1073/pnas.1010364107 21098262 PMC3003085

[88] Al-Awqati Q. 2007 Homer W. Smith award: control of terminal differentiation in epithelia. J Am Soc Nephrol. 2008;19(3):443–9. 10.1681/ASN.2007111195 18199795

[89] Trapnell C, Roberts A, Goff L, Pertea G, Kim D, Kelley DR, et al Differential gene and transcript expression analysis of RNA-seq experiments with TopHat and Cufflinks. Nature protocols. 2012;7(3):562–78. 10.1038/nprot.2012.016 22383036 PMC3334321

[90] Jiang H, Wong WH. Statistical inferences for isoform expression in RNA-Seq. Bioinformatics. 2009;25(8):1026–32. 10.1093/bioinformatics/btp113 19244387 PMC2666817

