# Supplementary figures and images for: The Gene Expression Program for the Formation of Wing Cuticle in Drosophila
Source: PLoS Genet. 2016 May 27;12(5):e1006100. doi: 10.1371/journal.pgen.1006100 (PMC4883753; doi:10.1371/journal.pgen.1006100)

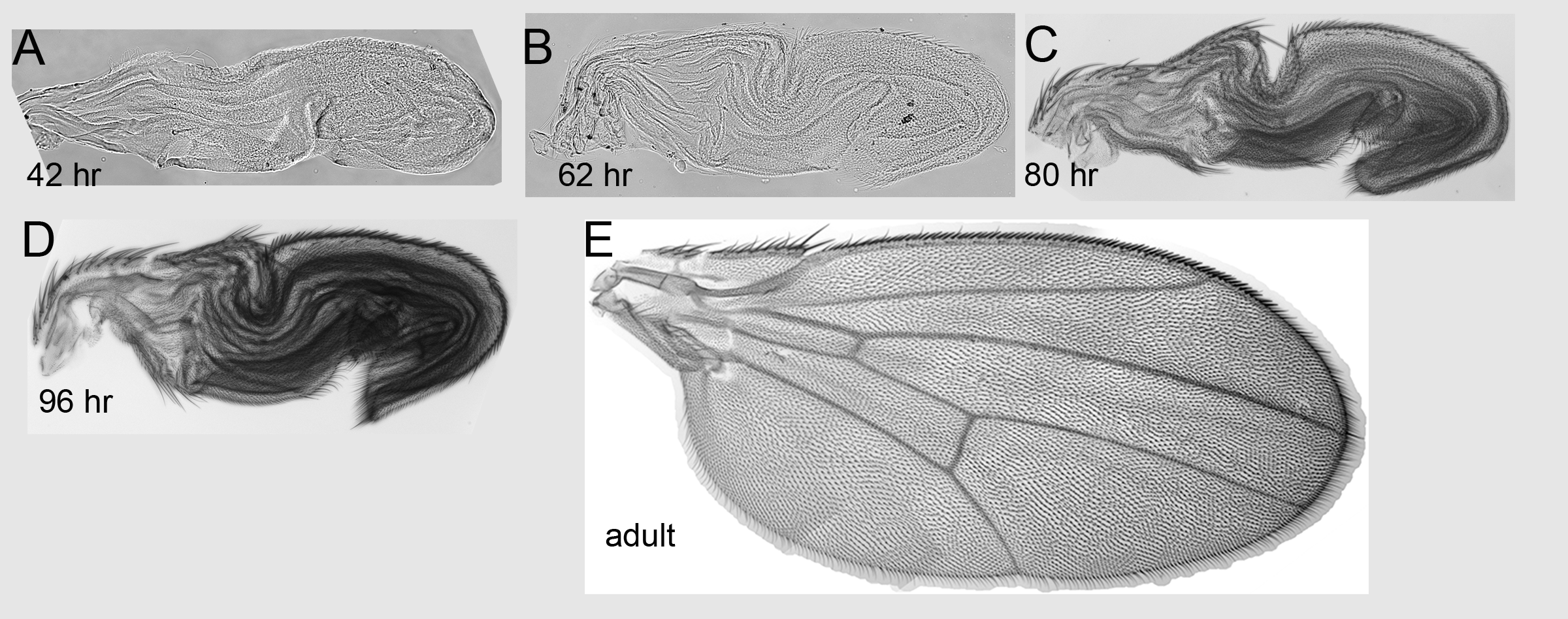

Supplement: S1 Fig — All images are light micrographs shown at the same magnification. Panel A– 42 hr pupal wing, B– 62 hr pupal wing, C– 80 hr pupal wing, D– 96 hr pupal wing and E an adult wing. For panels A and B the field diaphragm was partially shut to increase contrast. The time is for pupae collected as white prepupae and aged at 25°C. Note the pigmentation that becomes obvious at 80 hr. When relatively young wings are dissected they “relax” so that they are not as tightly folded back as they appeared in the pupae. (TIF) [file pgen.1006100.s001.tif]

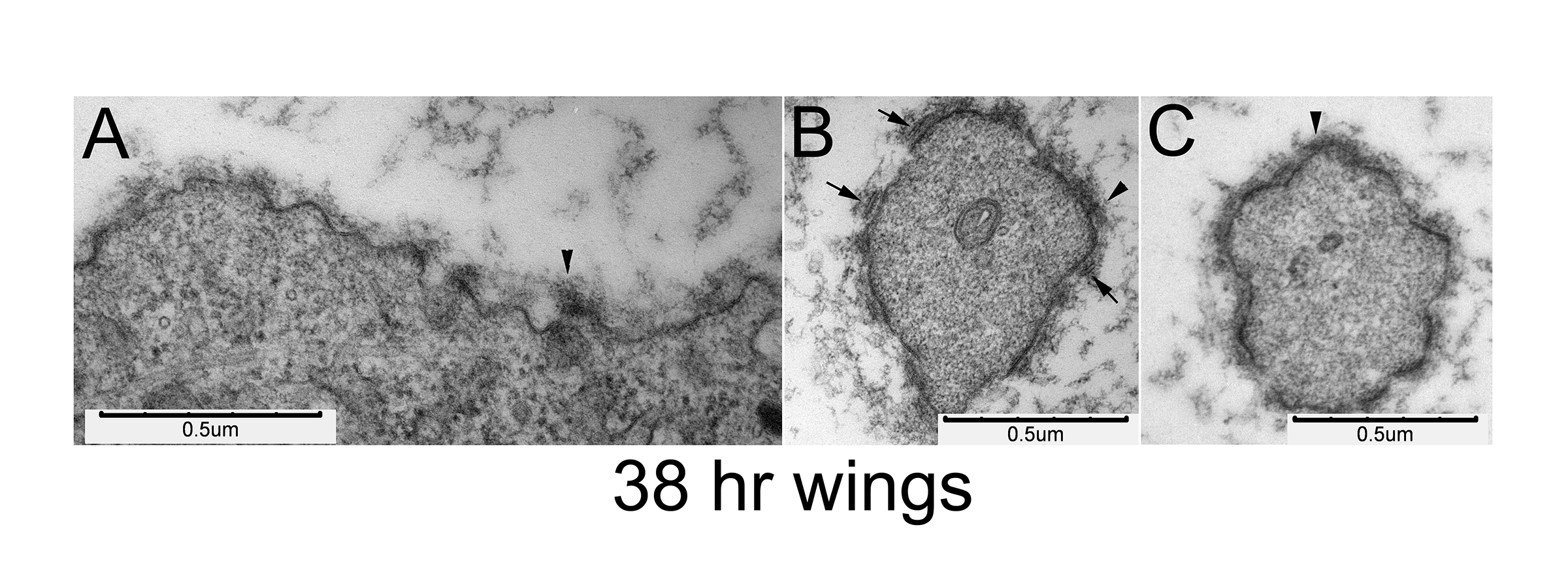

Supplement: S2 Fig — A shows the secretion of relatively electron dense material (arrowheads) from putative undulae in the region of future wing blade. B and C are cross sections of developing hairs. The arrows point to patches of typical envelope and the arrowheads to more amorphous electron dense material. B shows a number of patches of envelope while C does not. All of these images came from the same wing. (TIF) [file pgen.1006100.s002.tif]

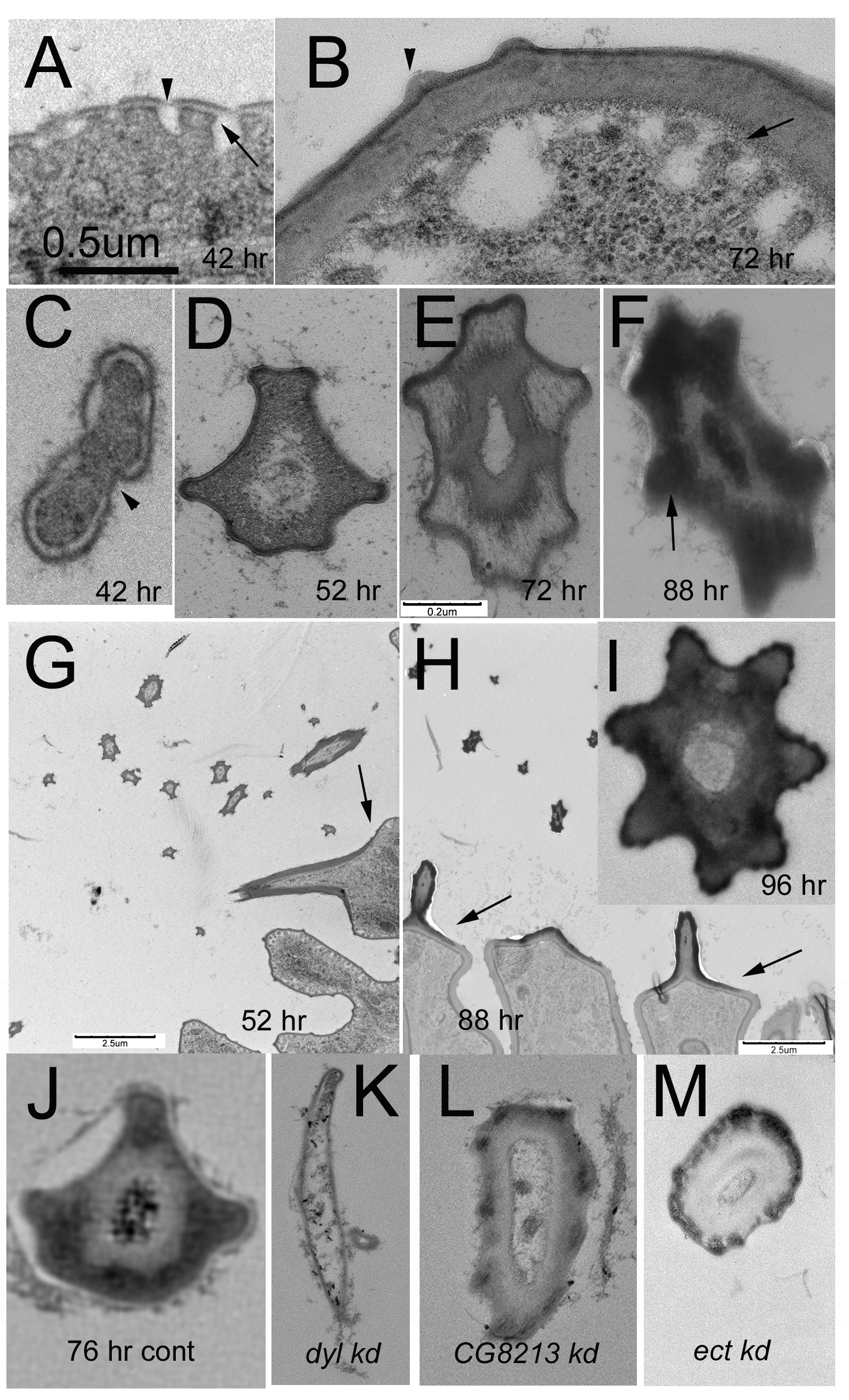

Supplement: S3 Fig — A. In 42 hr awp wings there are interruptions in the envelope (arrowhead) and cytoplasmic protrusions are seen where there is close contact between the envelope and the cytoplasm (arrows). The protrusions are thought to be sites of cuticle deposition B. Cytoplasmic protrusions (arrows) that are closely juxtaposed to the cuticle are also seen in later wings (72 hr awp). Both A and B at shown at the same magnification. Panels C-F and I are cross sections through developing hairs at progressively later stages. All of these images are shown at the same magnification. In panel C (42 hr) the developing envelope is incomplete with gaps present (arrowhead). By 52 hr awp (D) the hairs have taken on their fluted shape. Dark areas are prominent in the 88 and 96 hr awp hairs (arrow) (FI) that likely represent pigment deposition. G and H show glancing sections that show developing hairs present on pedestals (arrows). Note the hair pigmentation spreads into the pedestals. J-M are hairs from 76–80 hr wings where a 42 hr gene was kd using ap-Gal4. J is a control hair from the ventral surface of the same wing as K (dorsal surface of ap>dyl-RNAi). (TIF) [file pgen.1006100.s003.tif]

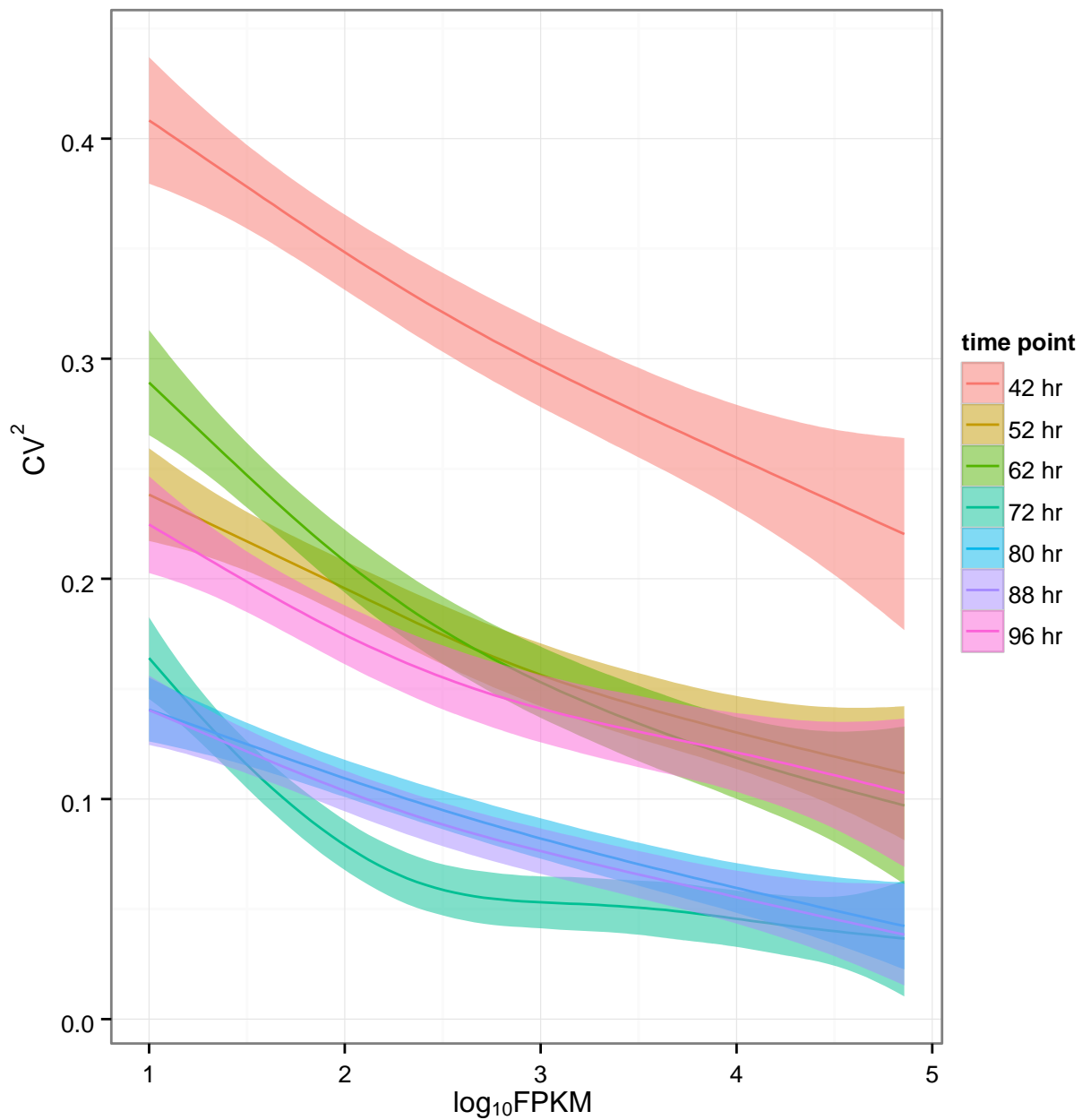

Supplement: S4 Fig — Plotted is the squared coefficient of variation (CV2) for genes with an expression level of FPKM≥10 as a function of log10FPKM. Note that the CV2 tended to decrease with increasing expression level and that the 42 hr sample showed the most variability. That sample was the most difficult to dissect and hence has the greatest chance of contamination by other cell types. (PDF) [file pgen.1006100.s004.pdf]
