# Supplementary material for: The Gene Expression Program for the Formation of Wing Cuticle in Drosophila
Source: PLoS Genet. 2016 May 27;12(5):e1006100. doi: 10.1371/journal.pgen.1006100 (PMC4883753; doi:10.1371/journal.pgen.1006100)
Supplement: S1 Table — (PDF) [file pgen.1006100.s005.pdf]

Table S1

Dorsal/Ventral cuticle thickness is altered in mutants

Genotype	Number of measurements	Number of segments	D/V ratio (sd)	p compared to wt*	V/D ratio (sd)	p compared to wt
Ore-R	120	6	1.23 (0.11)	-	0.82 (0.079)	-
dyl-i	140	7	2.08 (0.43)	0.0017	-	-
ect-i	160	8	1.79 (0.22)	8.2E-05	-	-
CG8213	180	9	-	-	2.29 (0.54)	2.9E-05

* t-test
