# Supplementary material for: The Gene Expression Program for the Formation of Wing Cuticle in Drosophila
Source: PLoS Genet. 2016 May 27;12(5):e1006100. doi: 10.1371/journal.pgen.1006100 (PMC4883753; doi:10.1371/journal.pgen.1006100)
Supplement: S3 Table — (PDF) [file pgen.1006100.s007.pdf]

Supplementary Table 3

Number of genes whose expression differs between time points.

	52 hr	62 hr	72 hr	80 hr	88 hr	96 hr
42 hr	1624	1750	2045	2310	2646	3067
52 hr		1266	1773	2085	2408	2939
62 hr			999	1626	2159	2839
72 hr				722	1611	2602
80 hr					1013	2352
88 hr						1639
