# Supplementary material for: The Gene Expression Program for the Formation of Wing Cuticle in Drosophila
Source: PLoS Genet. 2016 May 27;12(5):e1006100. doi: 10.1371/journal.pgen.1006100 (PMC4883753; doi:10.1371/journal.pgen.1006100)
Supplement: S6 Table — (PDF) [file pgen.1006100.s010.pdf]

Supplementary Table 6

The number of genes whose expression level (FPKM) at any time point is greater than 90 % (80%, 50%) of the sum of expression at all time points.

stage	# genes* >90% FPKM	# genes >80% FPKM	# genes >50% FPKM
42 hr	67	100	274
52 hr	34	61	167
62 hr	7	13	80
72 hr	1	2	23
80 hr	0	0	15
88 hr	0	0	26
96 hr	87	169	597

- * Only genes with a total FPKM > 10 were considered (7184 genes)
