# Supplementary material for: The Gene Expression Program for the Formation of Wing Cuticle in Drosophila
Source: PLoS Genet. 2016 May 27;12(5):e1006100. doi: 10.1371/journal.pgen.1006100 (PMC4883753; doi:10.1371/journal.pgen.1006100)
Supplement: S7 Table — (PDF) [file pgen.1006100.s011.pdf]

Supplementary Table 7

Phenotypes Associated with Genes in Clusters

Cluster #	Number of genes in cluster	Number annotated with a wing phenotype^	Number with RNAi phenotype@	# lethal@	# Bristle@	# Bristle morpho-logy@	# color@	# Notum@	# PCP@	# genes only in insects or arthropods
**1**	347	117 (.34)	89 (.26)	30 (.09)	50 (.14)	36 (.10)	17 (.05)	47 (.14)	10 (.03)	79 (.23)
**2**	347	125 (.36)	101 (.29)	47 (.14)	56 (.16)	43 (.12)	14 (.04)	47 (.14)	8 (.02)	95 (.27)
**3**	149	18 (.12)	25 (.17)	13 (.09)	18 (.12)	8 (.05)	5 (.03)	14 (.09)	2 (.01)	77 (.52)
**4**	860	319 (.37)	285 (.33)	164 (.19)	68 (.08)	14 (.02)	39 (.05)	102 (.12)	23 (.03)	129 (.15)
**5**	276	74 (.27)	60 (.22)	22 (.08)	31 (.11)	17 (.06)	12 (.04)	34 (.12)	3 (.01)	84 (.30)
**6**	832	271 (.33)	229 (.28)	119 (.14)	128 (.15)	94 (.11)	37 (.04)	124 (.15)	17 (.02)	153 (.18)
**7**	297	58 (.20)	63 (.21)	27 (.09)	38 (.13)	28 (.09)	8 (.03)	40 (.13)	5 (.02)	82 (.28)
**8**	162	28 (.17)	29 (.18)	13 (.08)	12 (.07)	9 (.06)	5 (.03)	10 (.06)	3 (.02)	97 (.60)
**9**	652	213 (.33)	227 (.35)	115 (.18)	118 (.18)	93 (.14)	24 (.04)	122 (.19)	20 (.03)	98 (.15)
**10**	555	179 (.32)	192 (.35)	105 (.19)	103 (.19)	81 (.15)	19 (.03)	98 (.18)	13 (.02)	99 (.18)
**11**	89	4 (.04)	10 (.11)	3 (.03)	7 (.08)	4 (.04)	3 (.03)	5 (.06)	0 (.00)	55 (.62)
**12**	89	7 (.08)	16 (.18)	4 (.04)	7 (.08)	6 (.07)	3 (.03)	8 (.09)	1 (.01)	55 (.62)
**13**	69	2 (.03)	7 (.10)	4 (.06)	6 (.09)	6 (.09)	0 (.00)	5 (.07)	0 (.00)	35 (.51)
**14**	200	33 (.17)	43 (.22)	22 (.11)	24 (.12)	17 (.09)	5 (.03)	23 (.12)	0 (.00)	80 (.40)
**15**	31	3 (.10)	5 (.16)	2 (.06)	2 (.06)	1 (.03)	0 (.00)	3 (.10)	0 (.00)	29 (.94)
**16**	140	18 (.13)	24 (.17)	9 (.06)	14 (.10)	9 (.06)	2 (.01)	17 (.12)	1 (.01)	71 (.51)
**total**	5095	1469 (.29)	1405 (.28)	699 (.14)	682 (.13)	466 (.09)	193 (.04)	699 (.14)	106 (.02)	1318 (.26)
**Chi-sq+**		$2X10^{-23}$	$4.4X10^{-11}$	$1.2X10^{-11}$	$3.5X10^{-7}$	$9X10^{-17}$	0.69	$5.3X10^{-4}$	0.29	$4.2X10^{-81}$

^ - data from FlyBase

@ - reference 42 (Mummery-Widmer JL, Yamazaki M, Stoeger T, Novatchkova M, Bhalerao S, Chen D, et al. Genome-wide analysis of Notch signaling in Drosophila by transgenic RNAi. (Nature. 2009;458(7241):987-92.) – For the bristle and notum columns all of the phenotypes in described in that paper were considered.

(fraction of total number of genes in that cluster showing the phenotype)

+ - a chi square test was used to determine if the observed number of genes showing a phenotype was different from that expected if all clusters had an equal frequency of genes producing the phenotype. P values are given.
