# Supplementary material for: The Gene Expression Program for the Formation of Wing Cuticle in Drosophila
Source: PLoS Genet. 2016 May 27;12(5):e1006100. doi: 10.1371/journal.pgen.1006100 (PMC4883753; doi:10.1371/journal.pgen.1006100)
Supplement: S8 Table — (PDF) [file pgen.1006100.s012.pdf]

Table S8

Gene ontology analysis of expression clusters.

cluster	silhouette (bigger is better)	overrepresented	underrepresented
1	-0.013	actin assembly, muscle attachment	metabolism
2	0.018	neuronal genes/cuticle pattern formation	transport
3	-0.087	structural components of chitin-based cuticle, extracellular matrix	metabolism
4	0.277	metabolism/housekeeping: translation, cell replication, cellular respiration	structural components of chitin-based cuticle
5	-0.068	transport, signaling, apoptosis	metabolism
6	0.174	secretion, vesicle transport, fusion, endosomes	translation, metabolism
7	0.004	active transport?	translation, metabolism
8	0.115	cell membrane associated?	translation, metabolism
9	0.186	chromatin remodeling, transcription	
10	0.121	protein folding, proteasome, cytoskeleton	transcription, translation
11	0.246	iron/heme binding, odorant binding	metabolism
12	0.014	no suggestions from GO (genes with expression pattern similar to CG10005)	metabolism
13	0.095	no suggestions from GO (yellow)	metabolism
14	-0.027	structural components of chitin-based cuticle	metabolism
15	0.369	plasma membrane, (Osiris family)	metabolism
16	0.125	no suggestions from GO (ple: genes important in cuticle maturation?)	metabolism
all significant	0.121	tube size – tracheal system, adherens junction, basement membrane, negative regulation of autophagy	nuclear genes, odorant binding, sensory perception, ribosome
