# Supplementary material for: The Gene Expression Program for the Formation of Wing Cuticle in Drosophila
Source: PLoS Genet. 2016 May 27;12(5):e1006100. doi: 10.1371/journal.pgen.1006100 (PMC4883753; doi:10.1371/journal.pgen.1006100)
Supplement: S1 File — (DOCX) [file pgen.1006100.s019.docx]

**Supplemental Methods**

**RNA isolation and library construction**

Pupal wings were dissected using a microscalpel and forceps, rinsed in ice-cold PBS, removed from pupal sacs and frozen at -80 ̊C in RNeasy Buffer RLT. Wings were thawed and kept on ice for additional collection and freezer storage time never exceeded two weeks. RNA was isolated from dissected wings using the RNeasy Mini Kit (Qiagen) and QIAcube. It was diluted to 500-1500 pg/ul and an accurate measure of its concentration determined by running on an Agilent Pico Chip. 1 μg of RNA was used to create unstranded RNA libraries using the NEBNextR Ultra™ RNA Library Prep Kit for IlluminaR according to the manufacturer’s instructions. The samples were multiplexed for an average read depth of ~8 million reads per sample on an Illumina MiSeq machine. Biological replicate libraries were sequenced and most of the data reported is on the average values of the duplicate samples. The concentration of the RNA library and the sequencing were done at the University of Virginia Biology Department Genomics Core.

**RT-qPCR confirmation of RNA-seq data**

Primers specific to all or selected transcripts were designed using Primer-BLAST76 and Geneious (Biomatters Ltd.). Whenever possible, one of the primers spanned exon-exon junctions to increase specificity to mRNA. 500 ng of RNA used in the second replicate run of RNA-seq was reverse transcribed into cDNA with SuperScriptR VILO MasterMix. The calibrator sample was made by adding together a volume of RNA from each of the seven samples so that the mass of each one was 1/7 of 500 ng and reverse transcribing the sample in the same way. Negative RT control sample was made by performing the reverse transcription protocol using a previously denatured reverse transcriptase. The RT-qPCR was done on an Applied Biosystems 7500 Fast Real Time PCR System. The reactions were set up using SYBR Select Master Mix (Life Technologies). SYBR GreenER Dye was the reporter dye. PCR cycling was done according to the kit protocol. 10 ng each template (library) was used for PCR cycling.

Two initial candidate endogenous control genes were selected: *RpL27A* (a commonly used gene – ribosomal protein) and *Xbp1* (an gene rather evenly expressed across time points as judged from RNAseq data). In final paper we used *Xbp1* because of its intermediate-level, stable expression and slightly higher primer efficiency (94.0% as opposed to 91.5%).

Additional information about RT-qPCR procedure (primer sequences, specificities, amplicon lengths, melt curves, standard curves, library concentrations) can be found in Supplementary File 4. The archive contains a readme file explaining the contents of each file.

**Sequencing runs details**

Library 1: 12 PCR cycles, 4 samples: 42 hr, 52 hr, 62 hr, 72 hr.

Library 2: 9 PCR cycles, 4 samples: 72 hr, 80 hr, 88 hr, 96 hr.

Library 3: 8 PCR cycles, 6 samples: 42 hr, 52 hr, 62 hr, 80 hr, 88 hr, 96 hr.

Library 1 sequencing run details: 100 bp paired-end using Illumina MiSeq v2 Kit, 25 M reads.

Libraries 2 and 3: 75 bp paired-end using Illumina MiSeq v3 Kit, 50 M reads.

Fragment length: no size selection on gel, estimated from Agilent Pico Chip results to ~400. RNA Integrity number (RIN) was found to be between 5.2 and 7.7 (undetermined for samples used to create Library 2 because of chip overloading).

**Analysis of RNA-seq data**

The adapter sequences needed for sequencing on the Illumina MiSeq platform were automatically trimmed by the software on BaseSpace website where all the data were deposited. FastQC78 was used to determine the overall quality of sequencing and to check for possible biases introduced by the library generation protocol. The Tuxedo RNA-seq analysis suite was used as described by the authors of the suite (1). Between 79.5 and 89.4% of the reads in the 14 samples were successfully mapped to the Drosophila genome sequence indicating that the sequence was of high quality. Downstream analysis of differential expression results, including production of heat map graphs, was done in R with the help of package cummeRbund and its dependencies. Terminal scripts used are available from Lukasz Sobala upon request. Gene clustering was done using a k-means algorithm (partitioning around medoids) and the optimal number of clusters was selected by maximizing the average cluster silhouette. A silhouette is a statistical measure of how well each datum, in this case expression pattern, sits within the cluster. Silhouette can assume values from -1 to 1, with 1 indicating prefect similarity between the datum and the cluster, and -1 indicating perfect dissimilarity. An average of these values is a measure of the cluster structure. Calculation of the average value across all clusters and plotting it against the number of clusters (k) was used to determine the optimal k. Expression data does not scale linearly but exponentially (highly expressed genes are several orders of magnitude more highly expressed than moderately expressed genes), therefore log_10_(FPKM + 1) values were used for clustering. The dendrogram (Fig. 3A) was produced using an algorithm measuring the distance (dissimilarity) between each data point. Other aspects of the analysis of differential expression were done in Excel. Novel transcript assessment was done in IGV (Integrated Genomics Viewer) (2, 3).

**RT-qPCR cycling parameters**

RT-qPCR plates (MicroAmp® Fast Optical 96-Well Reaction Plate) and plate adhesive covers (MicroAmp® Optical Adhesive Film Kit) were provided by Applied Biosystems.

Pre-cycling: Uracil-DNA Glycosylase (UDG activation step): 50 ̊C for 2 min, DNA Polymerase activation: 95 ̊C for 2 min.

Cycling: denaturation: 95 ̊C for 15 s, annealing/extension: 60 ̊C for 60 s. Repeated 40 times.

Dissociation: 95 ̊C for 15 s, 60 ̊C for 60 s.

Melt curve collection: increase temperature from 60 ̊C to 95 ̊C at a rate of 1 ̊C/min.

**Mapping of RNA-seq data**

Genome and annotations used: BDGP6 (FlyBase genome version 6.02). 17234 genes, 33634 isoforms, classical chromosomes and unaligned genome sequences included.

Command line options for Tophat (v2.0.12):
--no-novel-juncs (disable novel transcripts discovery for differential expression analysis).

Cuffdiff (v2.2.1) options used:
--frag-bias-correct (to the genome sequence provided);
--multi-read-correct;
--min-alignment-count 15 (filter out very sparsely expressed sequences from subsequent statistical tests);
--max-bundle-frags 999999999 (no upper limit on the number of fragments to include in statistical tests);
--no-effective-length-correction (corrections for size-selected libraries turned off to fix very short sequences with low counts being reported as having extremely high FPKM values).
Cuffdiff false discovery rate (FDR) left at default 0.05.

cummeRbund
(v2.8-2.12): statistical significance at 0.05.

**Isoform switching analysis:**

A custom R script was written to search for transcripts of one gene that fulfilled the following criteria:

- at least 2-fold change between time points;
- at least 50 FPKM for each isoform at the point of higher expression.

No statistical tests were performed on the results of this search.

**Uncertainty analysis**

The uncertainty in RT-qPCR RQ values is the standard deviation (sd) as reported by Applied Biosystems 7500 Fast RT-PCR System Software.

Cuffdiff uses a statistical model to infer the confidence intervals of FPKM values (4, 5)To obtain the theoretical FPKM value for a gene in the calibrator “pooled” sample, an arithmetic average of FPKMs in all 7 time points was calculated. We believe that without having the exact data from the sample, it is reasonable to assume the average. The uncertainty in the value was calculated as follows:

$$\sigma_{{FPKM}_{pooled}}=\frac{\sqrt{\sum_{n}^{7} \sigma_{X}^{2}}}{7}$$

Where:

- X – a gene of interest;
- n – a given sample;
- σ_X_ – 95% confidence interval for a given gene.

We assumed the uncertainty of expression of a given gene in the calibrator was uncorrelated with the uncertainty in each sample. To determine the 95% confidence intervals for data used to prepare Fig. 4 (expression relative to calibrator), the following error propagation formula was used:

$$\sigma_{RelX}=RelX\sqrt{\left( \frac{\sigma_{{FPKM}_{X}}}{{FPKM}_{X}} \right)^{2}+\left( \frac{\sigma_{{FPKM}_{pooled}}}{{FPKM}_{pooled}} \right)^{2}}$$

Where:

- X – a gene of interest in one sample;
- RelX – expression of the gene of interest relative to its expression in the calibrator sample $\left( =\frac{{FPKM}_{X}}{{FPKM}_{pooled}} \right)$
- σ_a_ – 95% confidence interval for a variable;
- FPKM_a_ – FPKM value for a variable.

**References**

1. Trapnell C, Roberts A, Goff L, Pertea G, Kim D, Kelley DR, et al. Differential gene and transcript expression analysis of RNA-seq experiments with TopHat and Cufflinks. Nature protocols. 2012;7(3):562-78.

2. Robinson JT, Thorvaldsdottir H, Winckler W, Guttman M, Lander ES, Getz G, et al. Integrative genomics viewer. Nature biotechnology. 2011;29(1):24-6.

3. Thorvaldsdottir H, Robinson JT, Mesirov JP. Integrative Genomics Viewer (IGV): high-performance genomics data visualization and exploration. Briefings in bioinformatics. 2013;14(2):178-92.

4. Trapnell C, Hendrickson DG, Sauvageau M, Goff L, Rinn JL, Pachter L. Differential analysis of gene regulation at transcript resolution with RNA-seq. Nature biotechnology. 2013;31(1):46-53.

5. Jiang H, Wong WH. Statistical inferences for isoform expression in RNA-Seq. Bioinformatics. 2009;25(8):1026-32.
