# Supplementary material for: The Gene Expression Program for the Formation of Wing Cuticle in Drosophila
Source: PLoS Genet. 2016 May 27;12(5):e1006100. doi: 10.1371/journal.pgen.1006100 (PMC4883753; doi:10.1371/journal.pgen.1006100)
Supplement: S3 File — (ZIP) [file pgen.1006100.s021.zip › Supplementary File 4 MIQE/MIQE_data_readme.docx]

**File: MIQE_library_data.xlsx**

The file contains a table summarizing the information about the generation of DNA libraries. Column “PCR cycles” refers to the number of PCR amplification cycles used to amplify the reverse transcribed, polyA-selected cDNA library. It is approximately inversely related to the resultant library concentration given in the rightmost column.

**File: MIQE_melt_curves.pdf**

The file contains melt curves for all primers used in RT-qPCR experiments. In the curve legends “pooled” refers to the library sample that was used in the experiment and the values to the right refer to its dilution. For NTC (No-Template Controls) no amplification template was provided. For –RT controls RNA libraries were used. Some NTC curves contain a low peak that signifies a product. The Cq in these wells was ~37, which suggests the starting DNA quantity is orders of magnitude less than 1 molecule. We do not have an explanation for it. These peak was not observed in any other melt curves which led us to conclude it is due to primer dimerization or miniscule contamination. Our results from RNAseq and qPCR agree very closely – we would not expect this if the samples had been contaminated. The primer for CG10005-RB was initially designed using a template sequence from the database. We noticed that our strain of Oregon-R fruit fly contains an SNP in the binding region of the forward primer. This explains the nonspecific, double curve. CG10005-RB (improved) shows the melt curve for the redesigned primer pair.

**File: MIQE_primer_data.xlsx**

A summary of information about used primers in a table form. Primer names are cross-referenced with titles of plots in MIQE_melt_curves.pdf. The file also contains data pertaining to the calculation of standard curves (i. e. lowest accepted dilution).

**File: MIQE_primer_standard_curves.pdf**

Standard curves for all used primers are found in this file. Linear fits were performed taking all three replicate samples into account. If the values diverged by more than 2% the data point was discarded. The abundance of CG10005-RA was very low which affected the accuracy of calculation of standard curve slope. For this primer we used an alternative technique and utilized Miner software (Sheng & Fernald, 2005) to estimate primer efficiency directly from amplification curves in other samples.

**References:**

Sheng Zhao, Russell D. Fernald. Comprehensive algorithm for quantitative real-time polymerase chain reaction. J. Comput. Biol. 2005 Oct;12(8):1045-62.
