# Supplementary material for: The Gene Expression Program for the Formation of Wing Cuticle in Drosophila
Source: PLoS Genet. 2016 May 27;12(5):e1006100. doi: 10.1371/journal.pgen.1006100 (PMC4883753; doi:10.1371/journal.pgen.1006100)
Supplement: S3 File — (ZIP) [file pgen.1006100.s021.zip › Supplementary File 4 MIQE/MIQE_melt_curves.pdf]

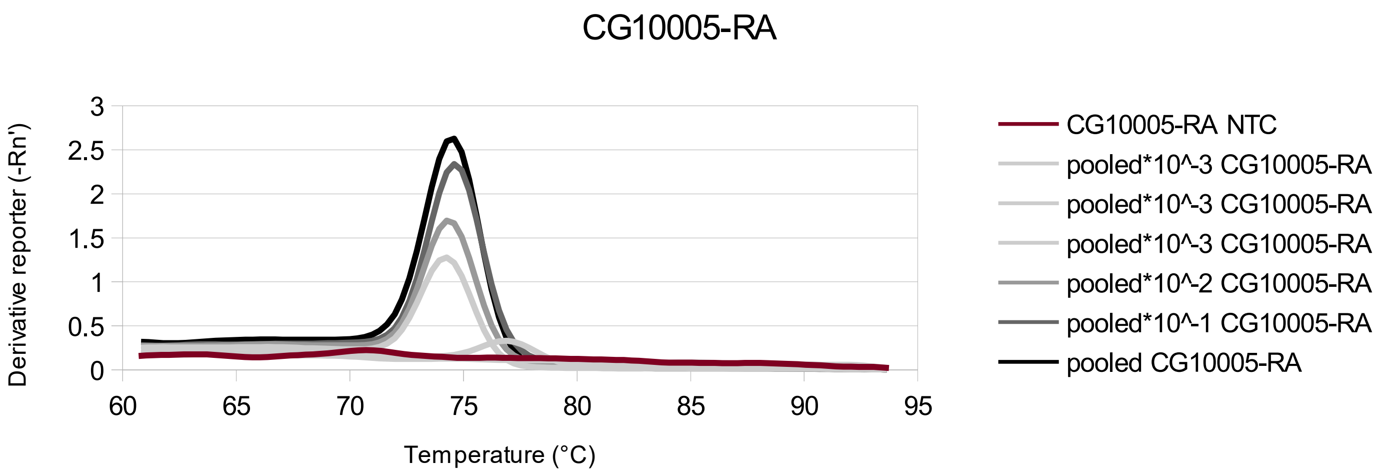
CG10005-RA
Derivative reporter (-Rn')
Temperature (°C)
0
0.5
1
1.5
2
2.5
3
60
65
70
75
80
85
90
95
CG10005-RA NTC
pooled*10^-3 CG10005-RA
pooled*10^-3 CG10005-RA
pooled*10^-3 CG10005-RA
pooled*10^-2 CG10005-RA
pooled*10^-1 CG10005-RA
pooled CG10005-RA

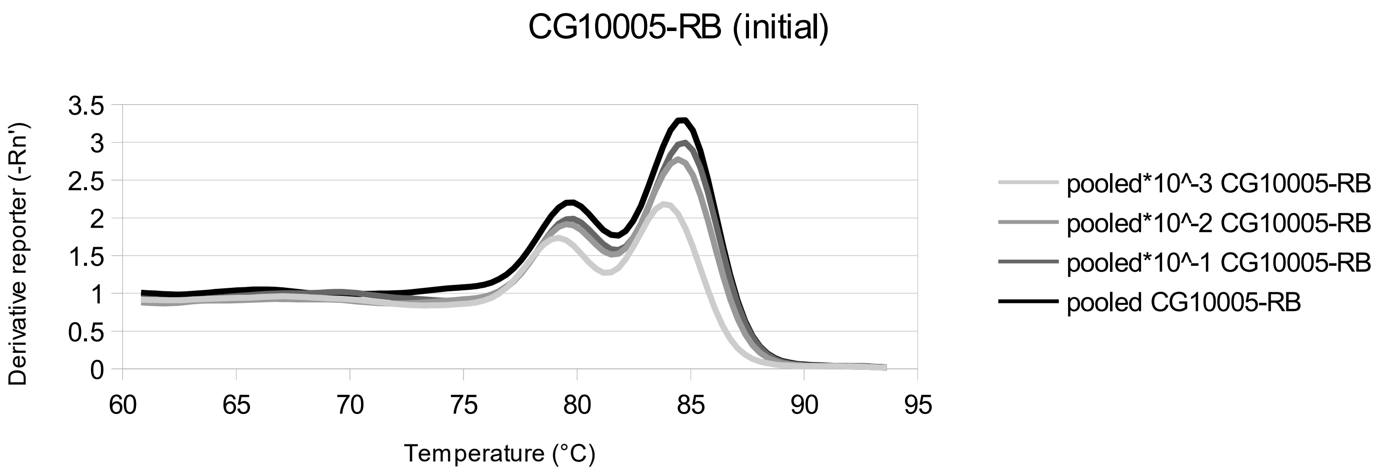
CG10005-RB (initial)
Derivative reporter (-Rn')
Temperature (°C)
0
0.5
1
1.5
2
2.5
3
3.5
60
65
70
75
80
85
90
95
pooled*10^-3 CG10005-RB
pooled*10^-2 CG10005-RB
pooled*10^-1 CG10005-RB
pooled CG10005-RB

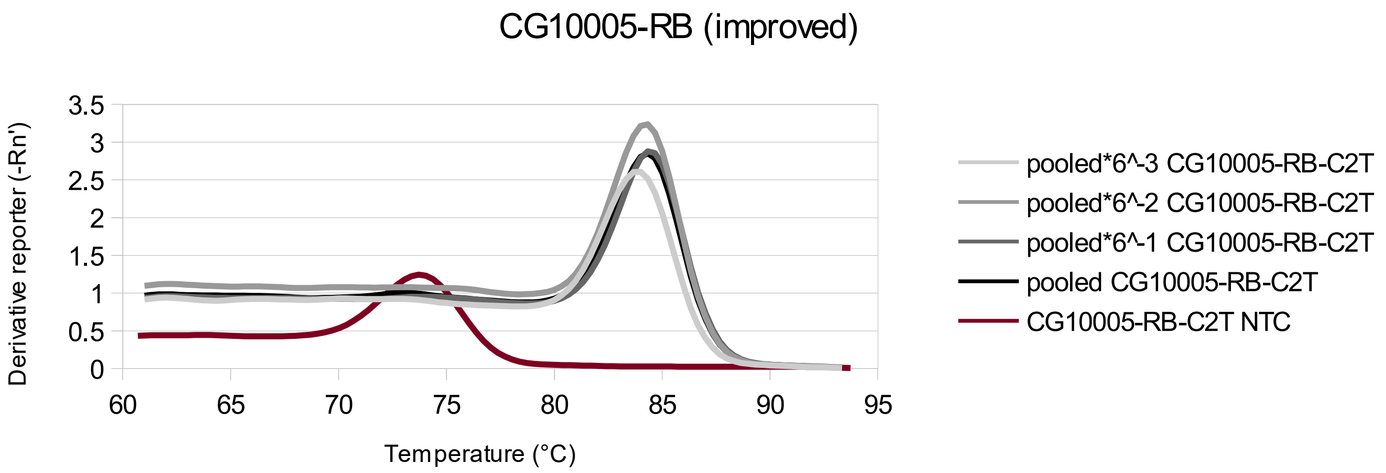
CG10005-RB (improved)
Derivative reporter (-Rn')
Temperature (°C)
0
0.5
1
1.5
2
2.5
3
3.5
60
65
70
75
80
85
90
95
pooled*6^-3 CG10005-RB-C2T
pooled*6^-2 CG10005-RB-C2T
pooled*6^-1 CG10005-RB-C2T
pooled CG10005-RB-C2T
CG10005-RB-C2T NTC

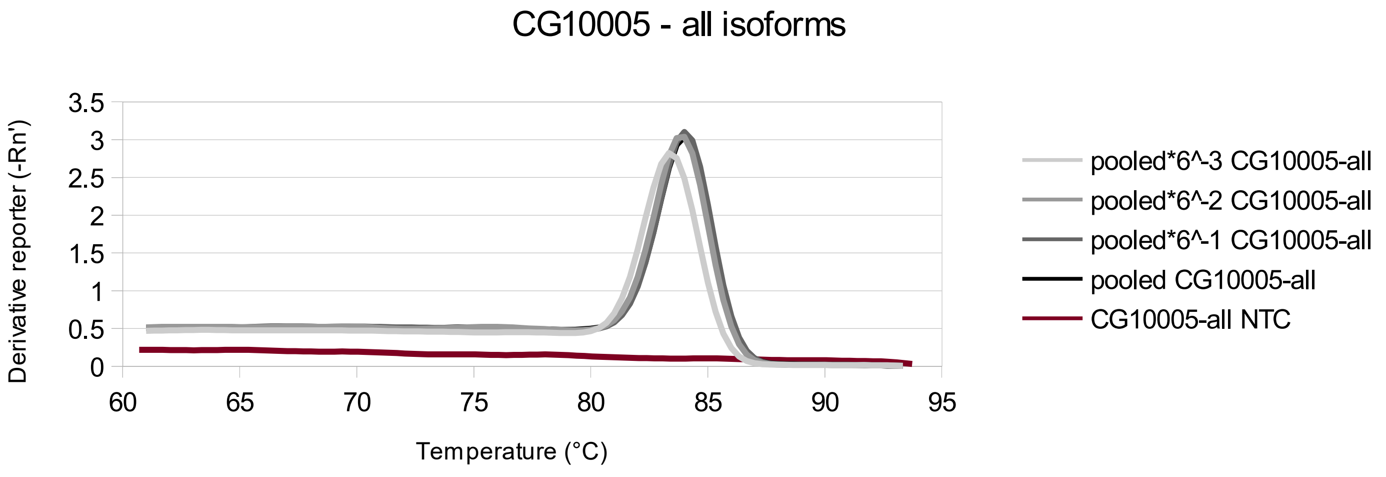
CG10005 - all isoforms
Derivative reporter (-Rn')
Temperature (°C)
0
0.5
1
1.5
2
2.5
3
3.5
60
65
70
75
80
85
90
95
pooled*6^-3 CG10005-all
pooled*6^-2 CG10005-all
pooled*6^-1 CG10005-all
pooled CG10005-all
CG10005-all NTC

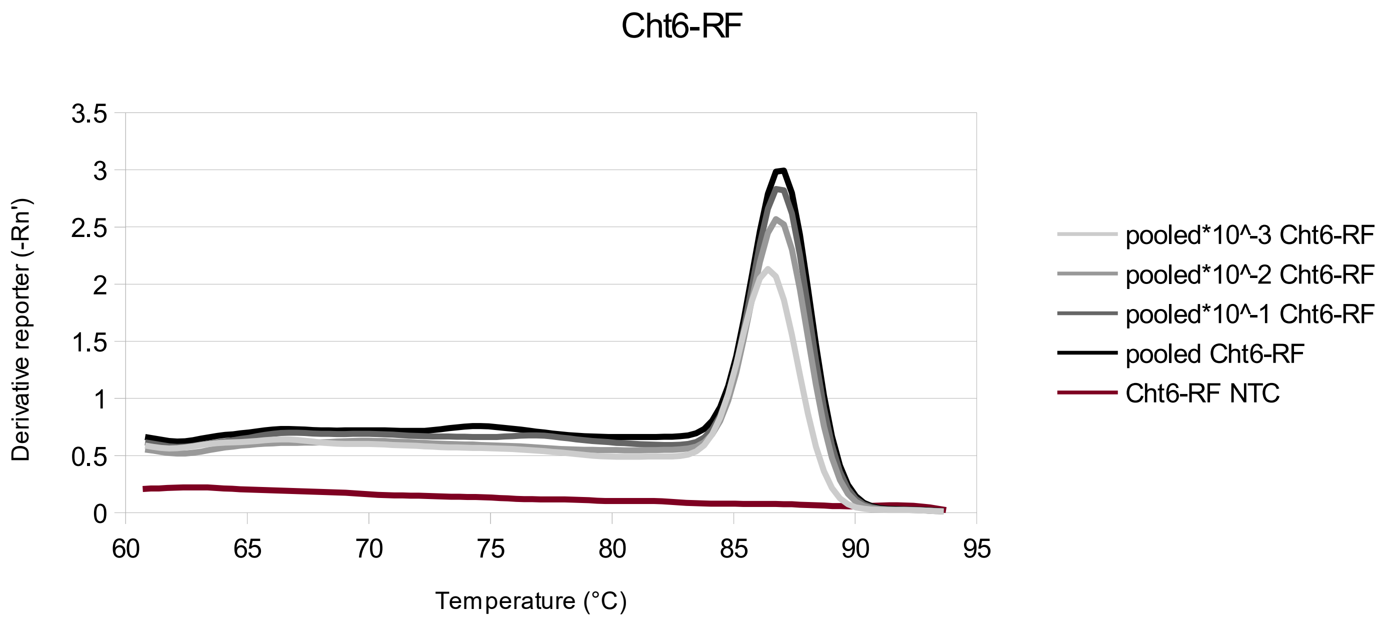
Cht6-RF
Derivative reporter (-Rn')
Temperature (°C)
pooled*10^-3 Cht6-RF
pooled*10^-2 Cht6-RF
pooled*10^-1 Cht6-RF
pooled Cht6-RF
Cht6-RF NTC

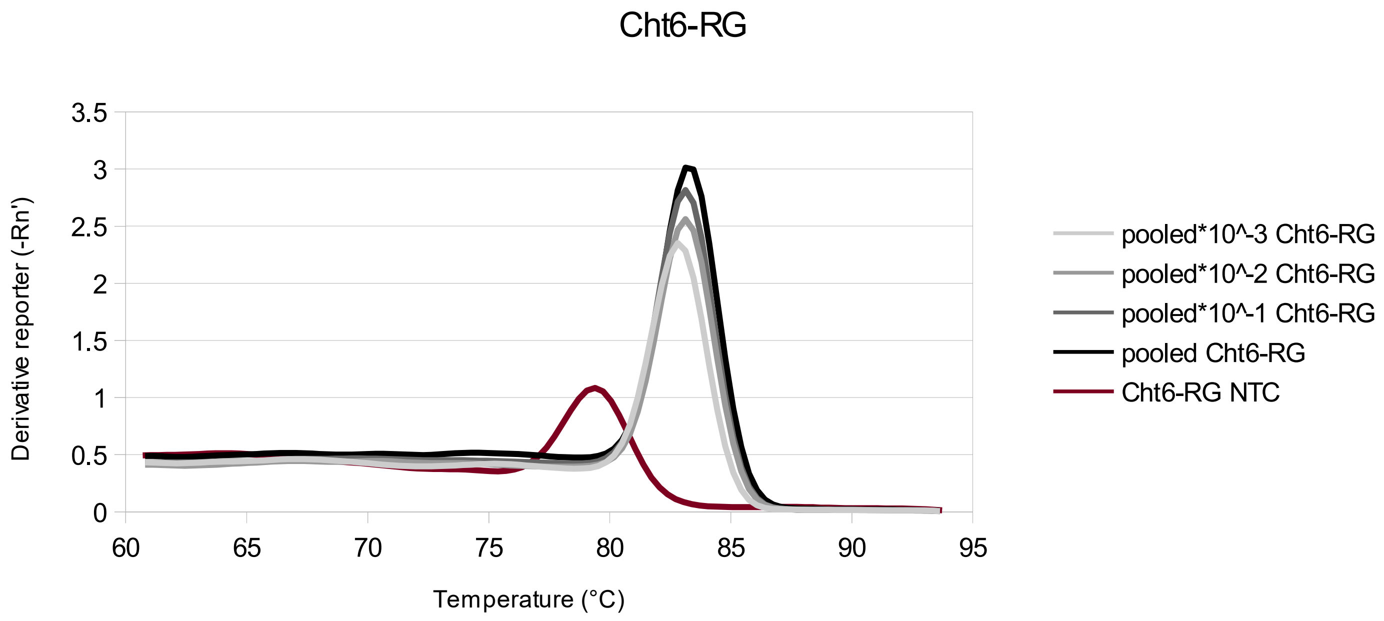
Cht6-RG
Derivative reporter (-Rn')
Temperature (°C)
pooled*10^-3 Cht6-RG
pooled*10^-2 Cht6-RG
pooled*10^-1 Cht6-RG
pooled Cht6-RG
Cht6-RG NTC

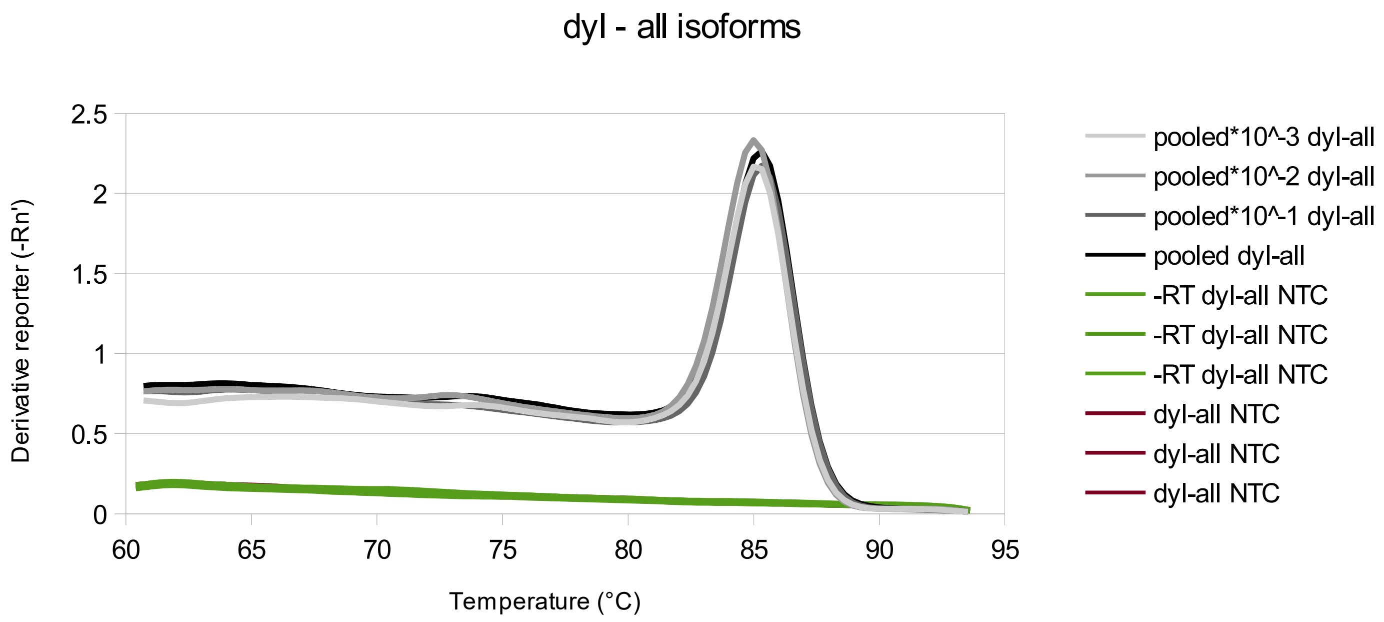
dyl - all isoforms
Derivative reporter (-Rn')
Temperature (°C)
pooled*10^-3 dyl-all
pooled*10^-2 dyl-all
pooled*10^-1 dyl-all
pooled dyl-all
-RT dyl-all NTC
-RT dyl-all NTC
-RT dyl-all NTC
dyl-all NTC
dyl-all NTC
dyl-all NTC

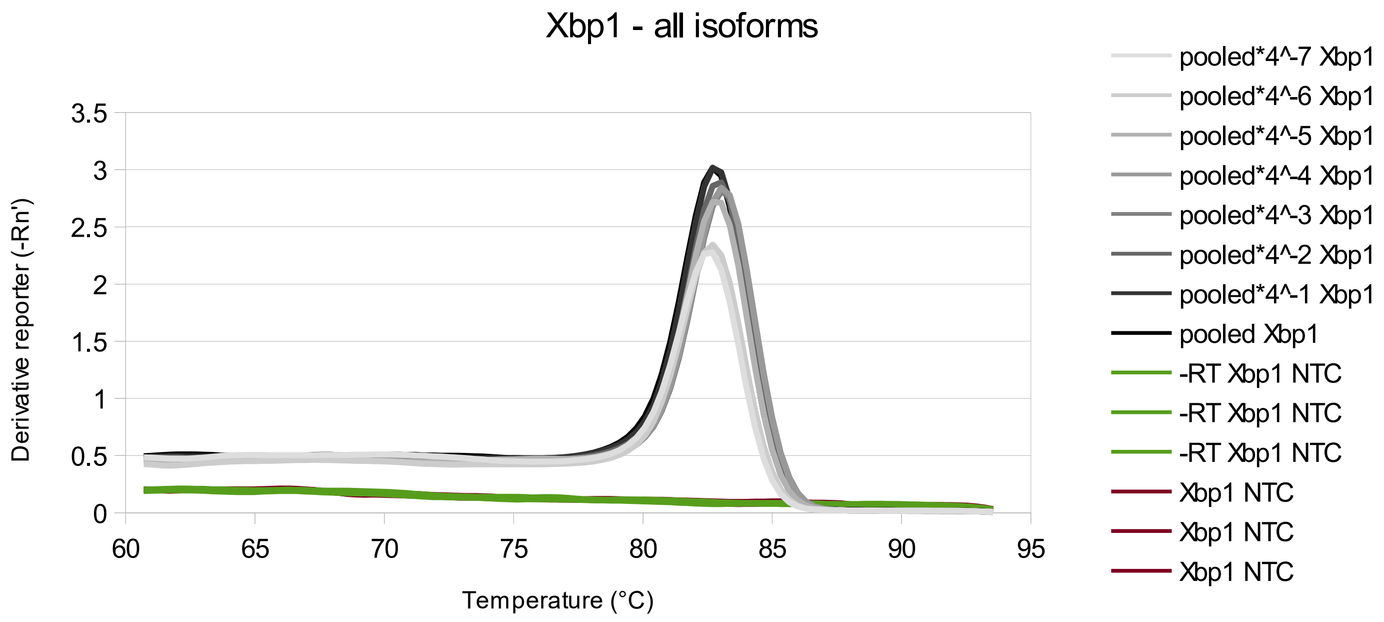

Xbp1 - all isoforms
Derivative reporter (-Rn')
3.5
3
2.5
2
1.5
1
0.5
0
60
65
70
75
80
85
90
95
Temperature (°C)
pooled*4^-7 Xbp1
pooled*4^-6 Xbp1
pooled*4^-5 Xbp1
pooled*4^-4 Xbp1
pooled*4^-3 Xbp1
pooled*4^-2 Xbp1
pooled*4^-1 Xbp1
pooled Xbp1
-RT Xbp1 NTC
-RT Xbp1 NTC
-RT Xbp1 NTC
Xbp1 NTC
Xbp1 NTC
Xbp1 NTC

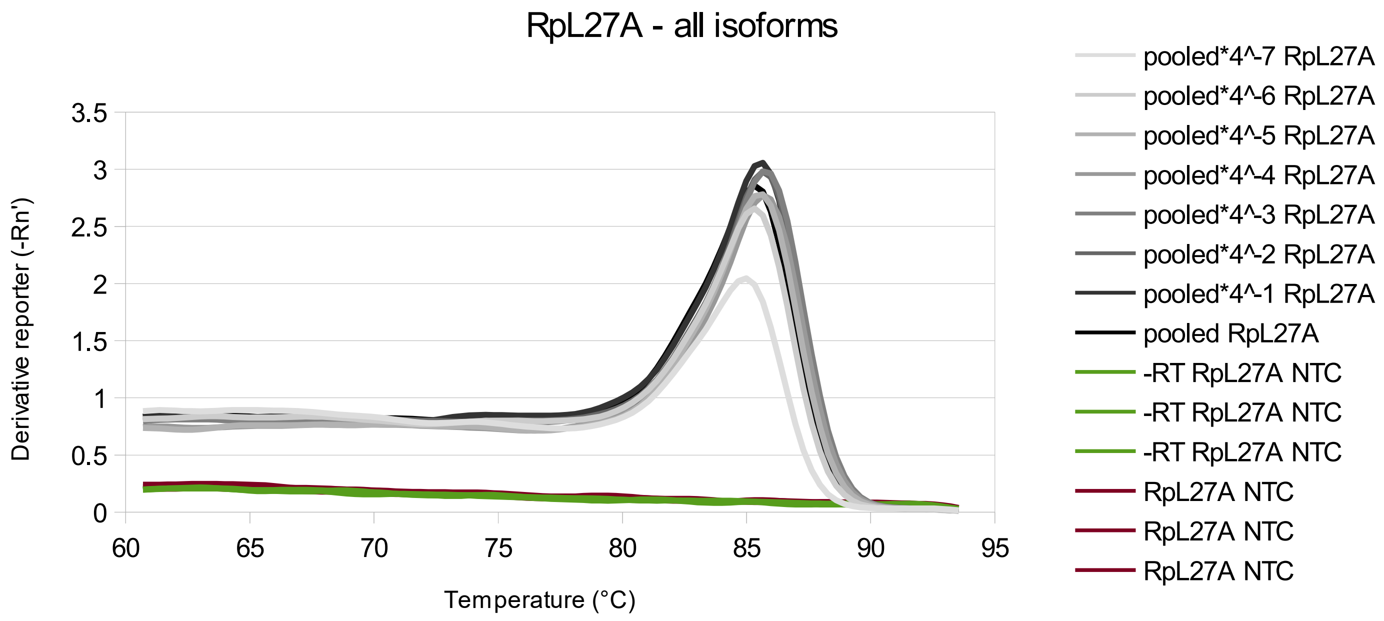

RpL27A - all isoforms
Derivative reporter (-Rn')
3.5
3
2.5
2
1.5
1
0.5
0
60
65
70
75
80
85
90
95
Temperature (°C)
pooled*4^-7 RpL27A
pooled*4^-6 RpL27A
pooled*4^-5 RpL27A
pooled*4^-4 RpL27A
pooled*4^-3 RpL27A
pooled*4^-2 RpL27A
pooled*4^-1 RpL27A
pooled RpL27A
-RT RpL27A NTC
-RT RpL27A NTC
-RT RpL27A NTC
RpL27A NTC
RpL27A NTC
RpL27A NTC

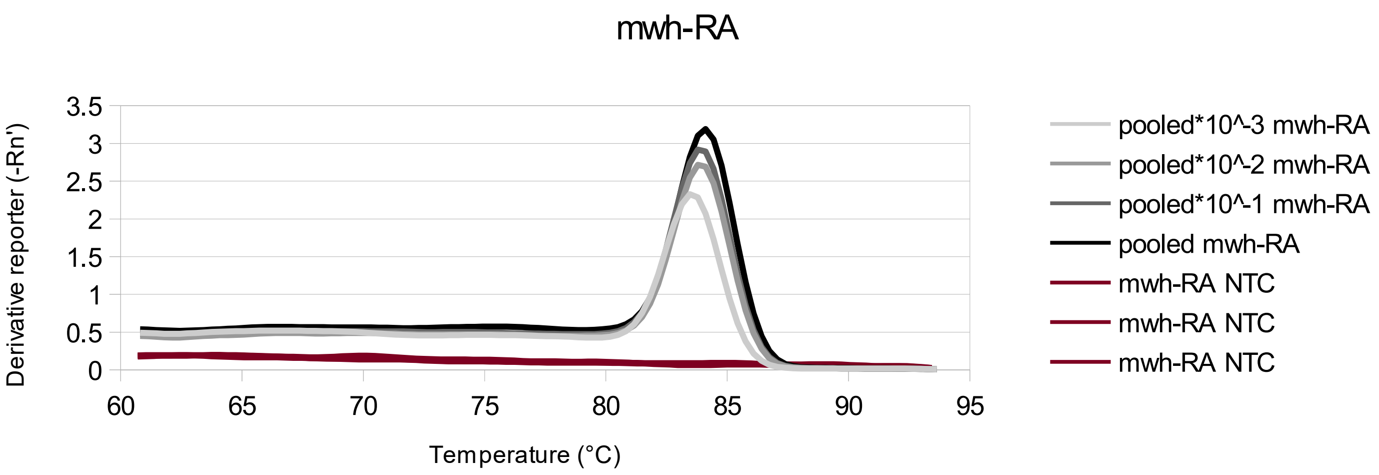
mwh-RA
Derivative reporter (-Rn')
3.5
3
2.5
2
1.5
1
0.5
0
60
65
70
75
80
85
90
95
Temperature (°C)
pooled*10^-3 mwh-RA
pooled*10^-2 mwh-RA
pooled*10^-1 mwh-RA
pooled mwh-RA
mwh-RA NTC
mwh-RA NTC
mwh-RA NTC

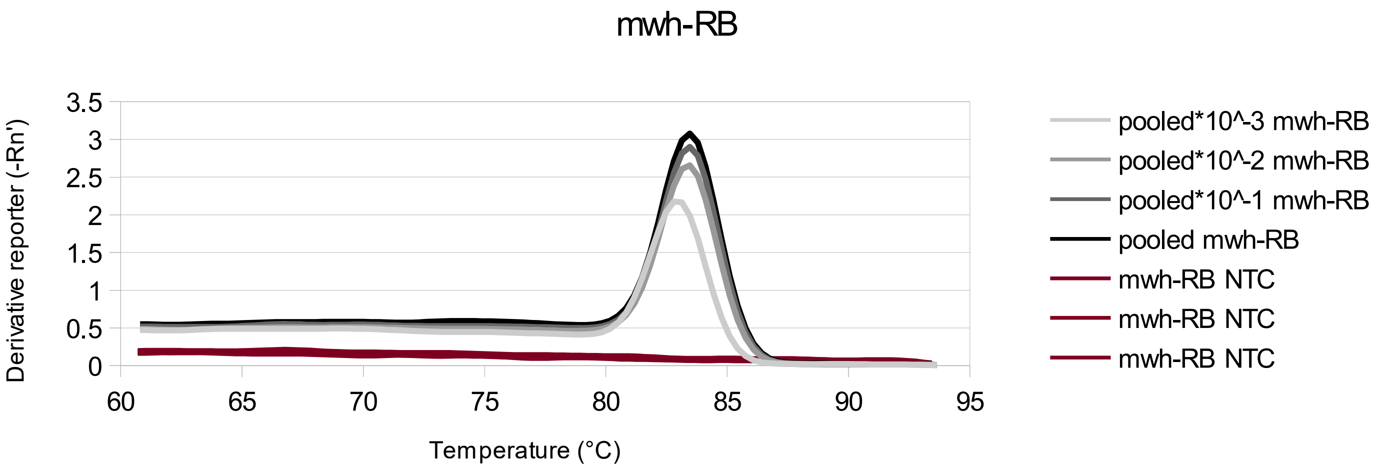
mwh-RB
Derivative reporter (-Rn')
3.5
3
2.5
2
1.5
1
0.5
0
60
65
70
75
80
85
90
95
Temperature (°C)
pooled*10^-3 mwh-RB
pooled*10^-2 mwh-RB
pooled*10^-1 mwh-RB
pooled mwh-RB
mwh-RB NTC
mwh-RB NTC
mwh-RB NTC

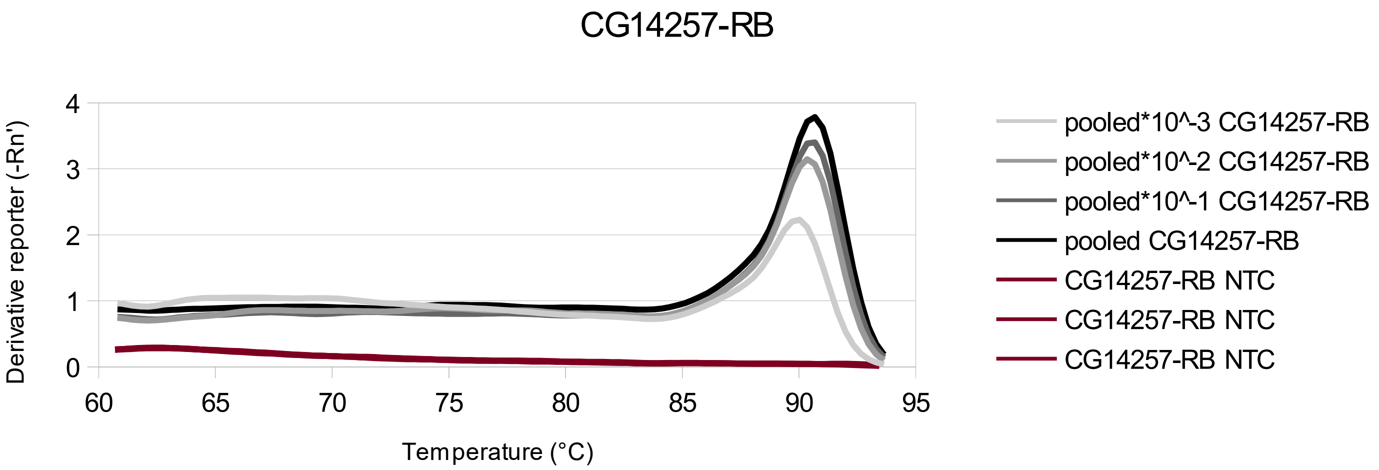
CG14257-RB
Derivative reporter (-Rn')
4
3
2
1
0
60
65
70
75
80
85
90
95
Temperature (°C)
pooled*10^-3 CG14257-RB
pooled*10^-2 CG14257-RB
pooled*10^-1 CG14257-RB
pooled CG14257-RB
CG14257-RB NTC
CG14257-RB NTC
CG14257-RB NTC

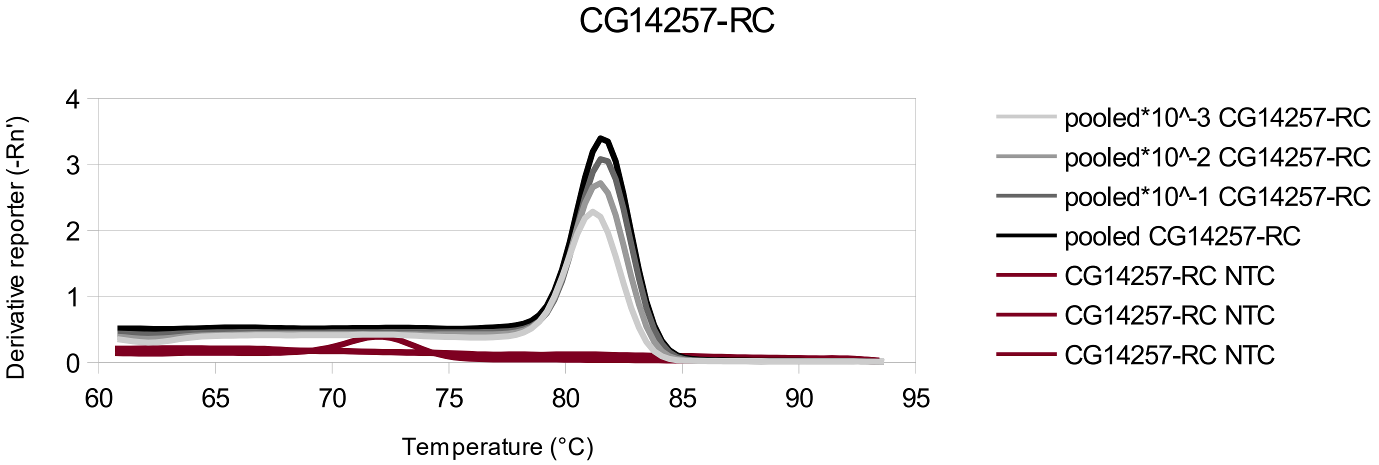
CG14257-RC
Derivative reporter (-Rn')
4
3
2
1
0
60
65
70
75
80
85
90
95
Temperature (°C)
pooled*10^-3 CG14257-RC
pooled*10^-2 CG14257-RC
pooled*10^-1 CG14257-RC
pooled CG14257-RC
CG14257-RC NTC
CG14257-RC NTC
CG14257-RC NTC
