# Supplementary material for: The Gene Expression Program for the Formation of Wing Cuticle in Drosophila
Source: PLoS Genet. 2016 May 27;12(5):e1006100. doi: 10.1371/journal.pgen.1006100 (PMC4883753; doi:10.1371/journal.pgen.1006100)
Supplement: S3 File — (ZIP) [file pgen.1006100.s021.zip › Supplementary File 4 MIQE/MIQE_primer_standard_curves.pdf]

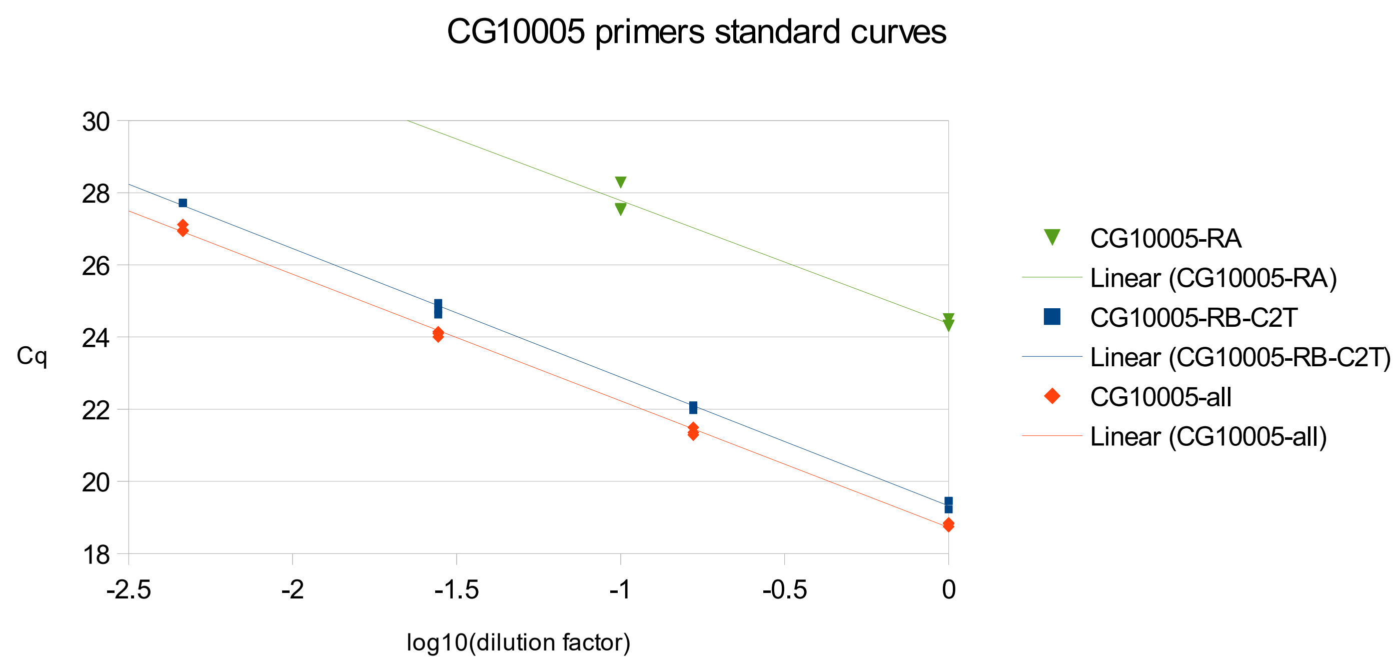
CG10005 primers standard curves
Cq
log10(dilution factor)
30
28
26
24
22
20
18
-2.5
-2
-1.5
-1
-0.5
0
CG10005-RA
Linear (CG10005-RA)
CG10005-RB-C2T
Linear (CG10005-RB-C2T)
CG10005-all
Linear (CG10005-all)

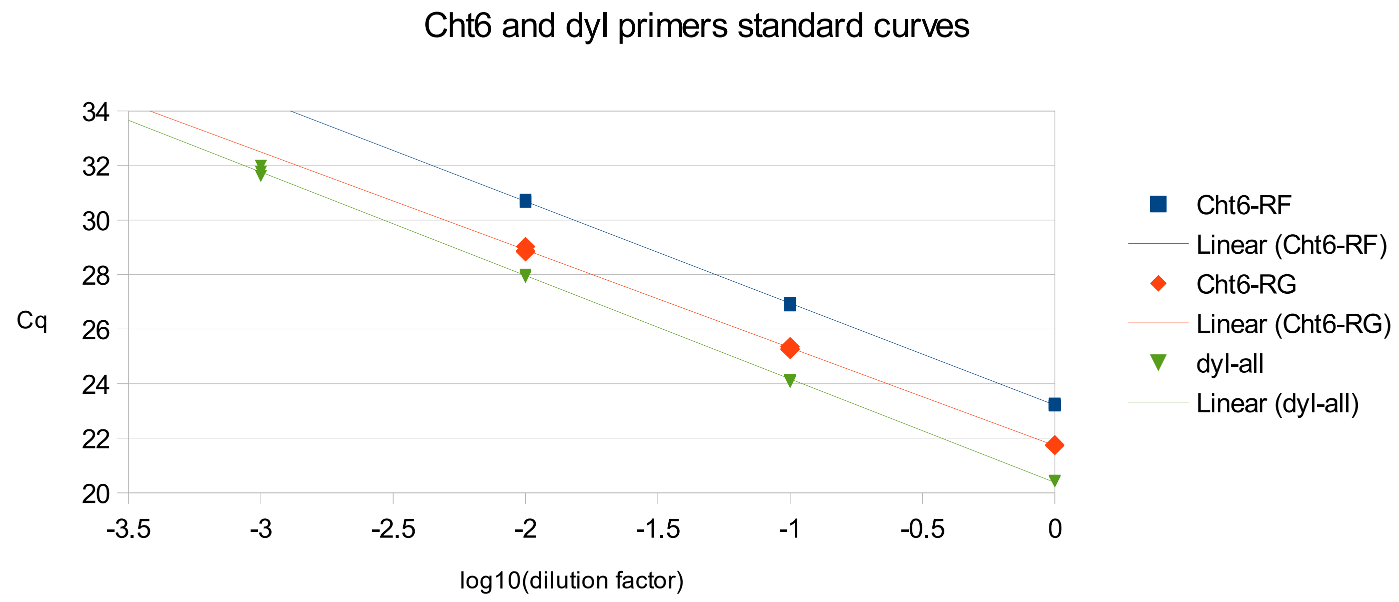
Cht6 and dyl primers standard curves
Cq
log10(dilution factor)
34
32
30
28
26
24
22
20
-3.5
-3
-2.5
-2
-1.5
-1
-0.5
0
Cht6-RF
Linear (Cht6-RF)
Cht6-RG
Linear (Cht6-RG)
dyl-all
Linear (dyl-all)

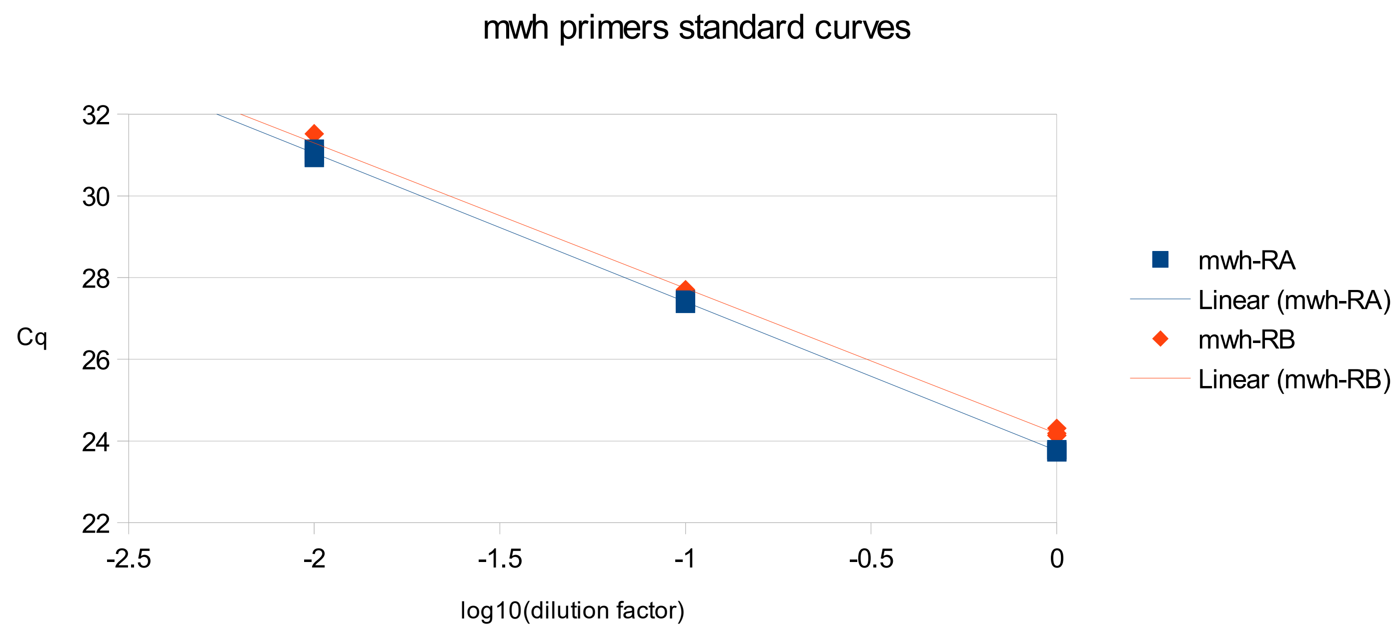
mwh primers standard curves
Cq
log10(dilution factor)
32
30
28
26
24
22
-2.5
-2
-1.5
-1
-0.5
0
mwh-RA
Linear (mwh-RA)
mwh-RB
Linear (mwh-RB)

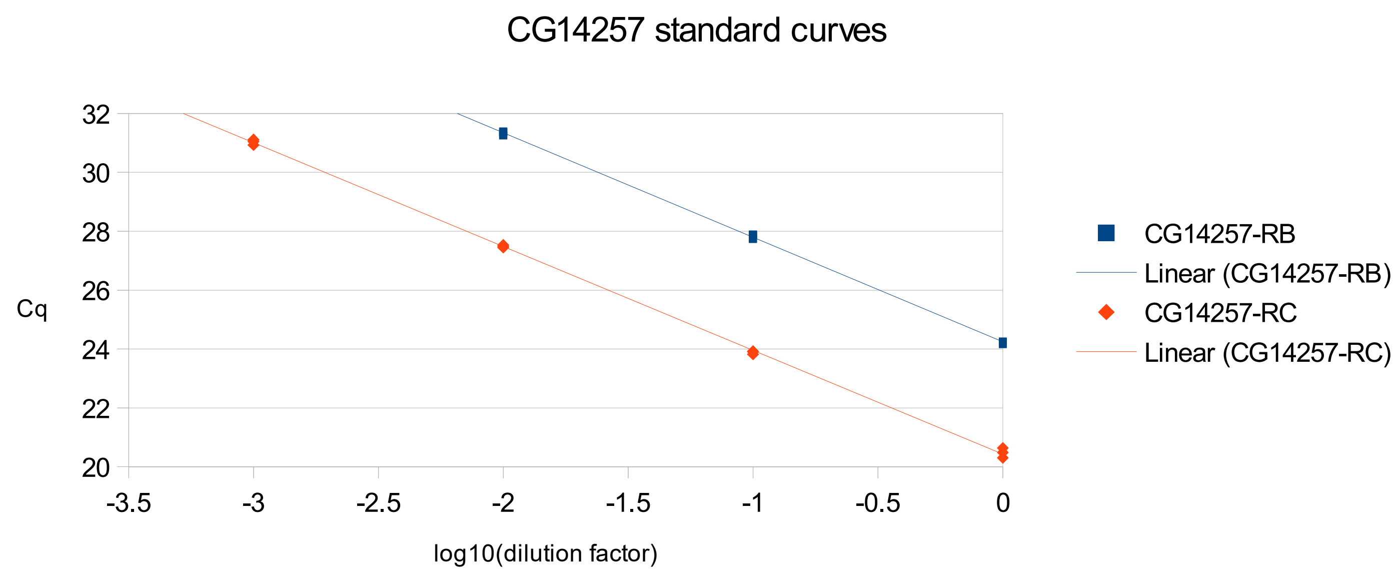
CG14257 standard curves
Cq
32
30
28
26
24
22
20
-3.5
-3
-2.5
-2
-1.5
-1
-0.5
0
log10(dilution factor)
CG14257-RB
Linear (CG14257-RB)
CG14257-RC
Linear (CG14257-RC)

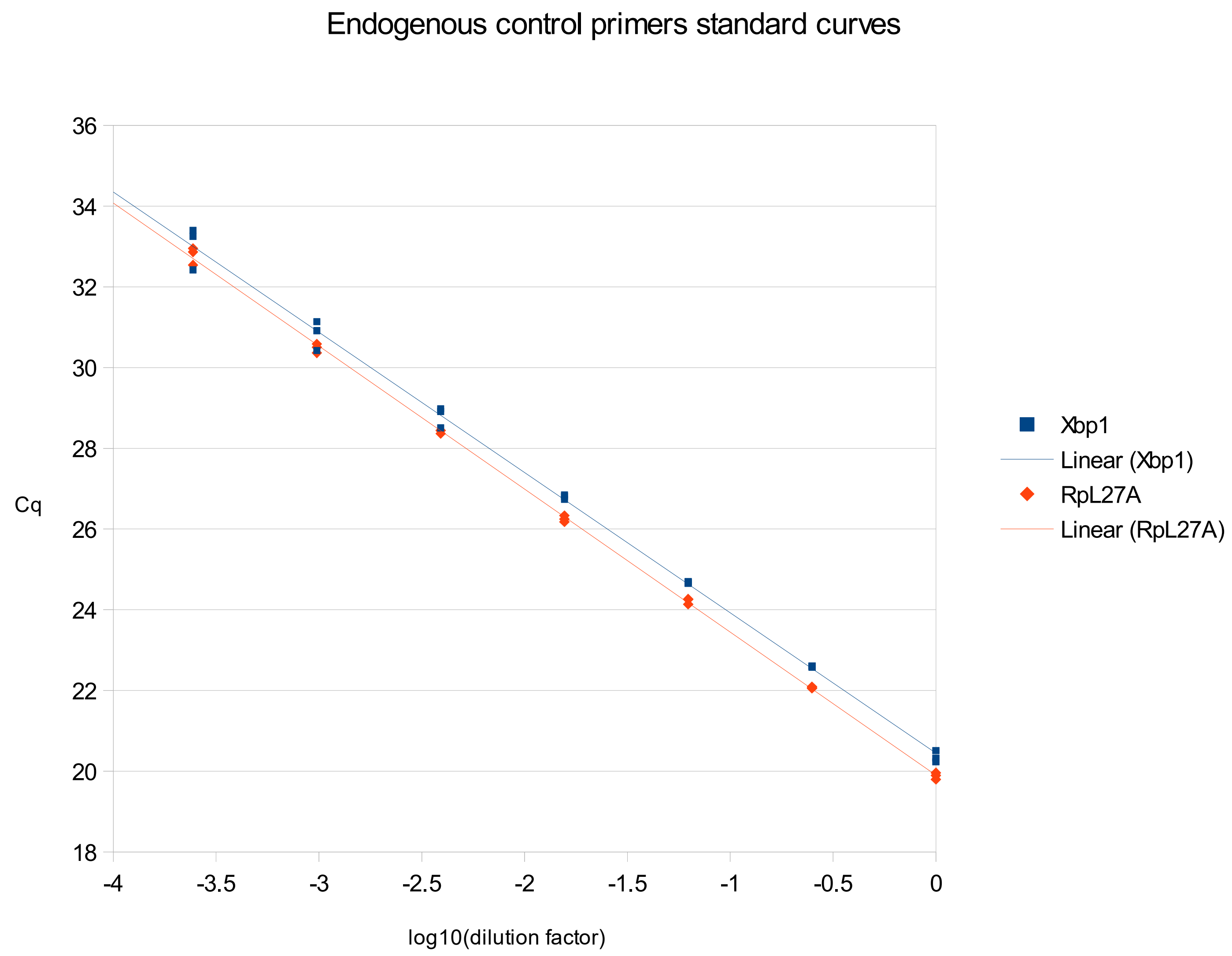
Endogenous control primers standard curves
Cq
36
34
32
30
28
26
24
22
20
18
-4
-3.5
-3
-2.5
-2
-1.5
-1
-0.5
0
log10(dilution factor)
Xbp1
Linear (Xbp1)
RpL27A
Linear (RpL27A)
